# Spatial identification of areas suitable for other effective area‐based conservation measures in the European Union

**DOI:** 10.1111/cobi.70263

**Published:** 2026-03-21

**Authors:** George Kefalas, Roxanne Suzette Lorilla, Stefanos Boutsios, Dimitrios Bormpoudakis, Pierre Bonnet, Alexandra Demertzi, Camino Liquete, Ioannis Ν. Vogiatzakis, Joseph Tzanopoulos, Jasmin Upton, Emily Howland, Heather Bingham, Konstantina Apostolopoulou, Neil Burgess, Evangelia G. Drakou

**Affiliations:** ^1^ Department of Geography Harokopio University Kallithea Greece; ^2^ Operational Unit BEYOND Centre for Earth Observation Research and Satellite Remote Sensing IAASARS, National Observatory of Athens Athens Greece; ^3^ Callisto Wildlife and Nature Conservation Society Thessaloniki Greece; ^4^ AMAP, Université de Montpellier, CIRAD, CNRS, INRA, IRD Montpellier France; ^5^ Department of Forest and Natural Environment Democritus University of Thrace Drama Greece; ^6^ Joint Research Centre (JRC) European Commission Ispra Italy; ^7^ Faculty of Pure and Applied Sciences Open University of Cyprus Nicosia Cyprus; ^8^ Department of Soil, Plant and Food Sciences University of Bari Aldo Moro Bari Italy; ^9^ Kent Interdisciplinary Centre for Spatial Studies University of Kent Canterbury UK; ^10^ Durrell Institute of Conservation and Ecology, Marlowe Building University of Kent Canterbury UK; ^11^ United Nations Environment Programme World Conservation Monitoring Centre (UNEP‐WCMC) Cambridge UK; ^12^ Centre for Macroecology, Evolution and Climate (CMEC) University of Copenhagen Copenhagen Denmark; ^13^ Department of Zoology University of Cambridge Cambridge UK

**Keywords:** biodiversity conservation, EU Restoration Law, Europe, OECMs, protected areas, Áreas protegidas, conservación de la biodiversidad, Europa, Ley de Restauración UE, OMCE, 其他有效区域性保护措施(OECMs), 保护区, 生物多样性保护, 《欧盟自然恢复法》, 欧洲

## Abstract

Although significant biodiversity has been safeguarded by protected areas (PAs), biodiversity trends continue downward. Within the frameworks of the EU Biodiversity Strategy for 2030 and the new EU Restoration Regulation (2024), conserving critical biodiversity areas is essential. One promising approach is other effective area‐based conservation measures (OECMs), which offer alternative pathways for in situ conservation. We identified sites across the EU that are not PAs but could contribute to conservation and be recognized as OECMs, and determined the potential contribution of these sites to biodiversity conservation. We partially applied the IUCN framework for OECM establishment across the entire EU with a focus on Greece and France. This framework is a three‐step process (screening, consent, and full assessment), the full application of which requires data related to the governance and management regime of the area, long‐term goals, and other site‐specific information. Such data are typically available only at the site‐based level. Hence, we based our analyses primarily on capturing biodiversity values within geographically defined areas based on freely available European data (e.g., landscape indices, habitats, and species statuses). We found that areas suitable for OECMs could cover up to 10% of European land and make substantial contributions to the EU's target of protecting 30% of its land by 2030. At the national level, we found that achieving biodiversity conservation outcomes with OECMs was uncertain. Accordingly, OECM confirmation must account for both the biodiversity value of proposed sites and the governance and management practices that support their effectiveness. Our approach highlights the potential of OECMs to enhance conservation efforts in support of a more resilient biodiversity landscape across Europe.

## Abstract (es)

Identificación espacial de zonas adecuadas para otras medidas de conservación eficaces basadas en zonas en la Unión Europea

**Resumen:**

Aunque las áreas protegidas (AP) han permitido salvaguardar una biodiversidad significativa, las tendencias en materia de biodiversidad siguen siendo negativas. En el marco de la Estrategia de Biodiversidad de la UE para 2030 y del nuevo Reglamento de la UE sobre restauración (2024), es esencial conservar las áreas críticas para la biodiversidad. Un enfoque prometedor son otras medidas de conservación eficaces basadas en áreas (OMCE), que ofrecen vías alternativas para la conservación in situ. Identificamos sitios en toda la UE que no son AP, pero que podrían contribuir a la conservación y ser reconocidos como OMCE, y determinamos la contribución potencial de estos sitios a la conservación de la biodiversidad. Aplicamos parcialmente el marco de la UICN para el establecimiento de OMCE en toda la UE, centrándonos en Grecia y Francia. Este marco es un proceso de tres pasos (selección, consentimiento y evaluación completa), cuya aplicación completa requiere datos relacionados con el régimen de gobernanza y gestión de la zona, los objetivos a largo plazo y otra información específica del lugar. Por lo general, estos datos solo están disponibles a nivel local. Por lo tanto, basamos nuestros análisis principalmente en la captura de los valores de biodiversidad dentro de áreas geográficamente definidas a partir de datos europeos de libre acceso (por ejemplo, índices de paisaje, hábitats y estado de las especies). Descubrimos que las áreas adecuadas para los OMCE podrían cubrir hasta el 10 % del territorio europeo y contribuir de manera sustancial al objetivo de la UE de proteger el 30 % de su territorio para 2030. A nivel nacional, descubrimos que el logro de resultados en materia de conservación de la biodiversidad con las OMCE era incierto. Por consiguiente, la confirmación de las OMCE debe tener en cuenta tanto el valor de la biodiversidad de los sitios propuestos como las prácticas de gobernanza y gestión que respaldan su eficacia. Nuestro enfoque destaca el potencial de las OMCE para mejorar los esfuerzos de conservación en apoyo de un paisaje de biodiversidad más resiliente en toda Europa.

## 欧盟境内适合实施其他有效区域性保护措施的区域空间识别

**【摘要】** 尽管保护区已保护了大量生物多样性, 但生物多样性持续下降的趋势仍未得到遏止。在《欧盟2030年生物多样性战略》及新版《欧盟自然恢复法》(2024)框架下, 保护关键生物多样性区域至关重要。其他有效区域性保护措施(other effective area‐based conservation measures, OECMs)作为一种有前景的方法, 为就地保护提供了替代路径。我们识别了欧盟范围内未被划为保护区, 但可贡献保护价值、且有潜力被认定为OECMs的位点, 并评估了这些位点对生物多样性保护的潜在贡献。我们在欧盟范围内部分应用了国际自然保护联盟(IUCN)的OECM建立框架, 重点关注希腊和法国。该框架包含三个步骤(筛选、同意和全面评估), 其完整应用需要关于区域治理及管理制度、长期目标, 以及其他位点特异性信息的数据, 而这些数据通常是位点水平的。因此, 我们的分析主要基于可公开获取的欧洲地区数据(如景观指数、栖息地与物种状况), 在地理界定的区域内评估生物多样性价值。研究发现, 适宜建立OECMs的区域可覆盖欧洲陆地面积的10%, 对欧盟实现“2030年前保护30%陆地”的目标具有重大贡献。在国家尺度上, 我们发现通过OECMs实现生物多样性保护目标仍存在不确定性。因此, OECMs的认定必须同时考虑拟定区域的生物多样性价值及支持其保护有效性所需的治理与管理实践。我们的方法强调了OECMs在加强保护工作方面的潜力, 来帮助在欧洲构建更具恢复力的生物多样性景观。**【翻译:胡怡思;审校:聂永刚】**

## INTRODUCTION

Global biodiversity is declining, with observed impacts on ecosystem services and human society (Pereira et al., 2024). Strategies, policies, and initiatives centered around protected areas (PAs) alone have safeguarded important biodiversity elements; however, they have not fully mitigated biodiversity loss (Díaz et al., 2019; Xu et al., 2021). The Kunming–Montreal Global Biodiversity Framework (KMGBF) (CBD, 2022) and the revised 2030 European Union (EU) Biodiversity Strategy (EC, 2020b) highlighted the need to enhance biodiversity protection through the expansion of area‐based conservation of marine, terrestrial, and freshwater realms to halt and reverse biodiversity decline. This raises the question of whether expanding or establishing PAs is the solution, particularly in cases where intense pressures often cause existing ones to fall short of their objectives (Liu et al., 2024; Mammides, 2020).

Target 3 of the KMGBF highlights the need to improve PA network quality alongside its expansion. This includes enhancing landscape connectivity, increasing ecosystem representation, and improving management effectiveness and equitable governance (CBD, 2022). Research on biodiversity governance to date has given one additional message: biodiversity conservation needs to go beyond PAs to be considered within global, regional, and local policies, including those not directly related to biodiversity (Maxwell et al., 2020; Gurney et al., 2021). Within this context, novel conservation approaches aim to identify areas with important biodiversity value (Jones et al., 2018; Kearney et al., 2022; Visconti et al., 2019) that are not yet protected under existing conservation efforts; these areas are called “other effective area‐based conservation measures (OECMs)” (IUCN, 2019).

Although PAs must aim “to achieve the long‐term conservation of nature with associated ecosystem services and cultural values” (Dudley, 2008), OECMs can have variable management objectives but must deliver effective conservation outcomes (CBD, 2018). OECMs provide an opportunity to recognize areas outside PAs that are managed for a range of purposes and under diverse governance regimes, where they contribute positively to biodiversity conservation. This can include areas managed and governed by government entities, Indigenous Peoples, local communities, a private group, or different sectors (e.g., archaeological sites, agricultural or hunting areas), operating under specific governance regimes based on sectoral needs and structures. Through their management, OECMs can simultaneously and sometimes unintentionally safeguard biodiversity (Belote & Wilson, 2020; Elleason et al., 2021). Under the recognition that biodiversity conservation needs to shift away from disciplinary and sectoral approaches (Gurney et al., 2021), the OECM concept, which embraces the role of other socioeconomic sectors operating in the same or adjacent space, offers a new tool for biodiversity conservation. OECMs can highlight the multiuse and multifunctionality of the natural environment, while considering sectoral and societal priorities (Visseren‐Hamakers et al., 2021).

To date, recognition of OECMs by Parties to the Convention on Biological Diversity (CBD) is an ongoing process, with only 16 countries having reported such areas to the World Database on OECMs (WD‐OECM). As of June 2025, OECMs cover 1.48 million km^2^ of terrestrial and 861,304 km^2^ of marine realms (UNEP‐WCMC & IUCN, 2025). In the EU, although the new European Biodiversity Strategy (BDS) (EC, 2020b) refers to the contribution of OECMs toward the achievement of its targets, we only detected a few attempts to describe those, whereas most policy efforts are devoted to the designation, effectiveness, and coherence of traditional PAs. To date, there are two studies that identify and assess OECMs at the EU level (Lázaro et al., 2021; Petza et al., 2019) and two at the national level for Spain (Rodríguez‐Rodríguez et al., 2021) and Cyprus (Vogiatzakis & Stavrinides, 2024). Yet, at the EU level, only Sweden has recognized and reported OECMs within the WD‐OECM (UNEP‐WCMC & IUCN, 2024).

Mapping OECMs is a significant step toward multisectoral biodiversity conservation. We aimed to identify suitable areas for terrestrial OECMs by applying IUCN's framework (which provides guidance on applying the CBD definition and criteria [CBD, 2018]), while assessing their biodiversity value. We had two main objectives: to propose and test a stepwise approach to map areas suitable for OECM establishment (i.e., areas that could be considered as OECMs if biodiversity value is confirmed and the full list of the IUCN criteria is formally validated), and to estimate the biodiversity value in these areas. To ensure scalability of the methodology, the analysis was conducted at the EU level and the national level for Greece and France. Based on the outcomes, the paper discusses the reproducibility of the approach across scales, the next steps toward the formal confirmation of OECMs, and the implications their establishment may have, in the context of the EU Green Deal (EGD) and the new EU Restoration Regulation.

## METHODS

### Spatial identification of OECM areas

To identify potentially suitable areas for OECMs at the EU27 and national levels, we utilized as a guide the IUCN's three‐step process: (Table 1): Step 1, screen; Step 2, consent; and Step 4, full assessment (Jonas et al., 2023).

**TABLE 1:** Criteria for screening sites and full assessment steps of the International Union for Conservation of Nature's guidance and data used in each step (adapted from Jonas et al. [2023]).

Criterion	Data used	Fulfilled		
		EU 27	Greece	France
Step 1: Screening assessment				
1.1: Site is not a protected area	Protected areas provided by WDPA	Yes	Yes	Yes
Criterion 1.2: Reasonable likelihood that the site supports important biodiversity values	Habitat and species (Article 17, 92/43/EEC and Article 12, 2009/147/EC) Forest landscape integrity index Landscape fragmentation index European roadless areas	Yes	Yes	Yes
Step 2: Consent for full assessment				
Criterion 2: Consent has been given to a potential OECM to carry out a full assessment	Criterion not assessed			
Step 3: Full assessment				
Criterion 3.1: Site is a geographically defined area	Criterion 1.2 Key biodiversity areas High nature value farmlands Greece Archaeological sites Forest Map France UNESCO geoparks National geological heritage sites Natural spaces Station verte villages	Yes	Yes	Yes
Criterion 3.2: Site is confirmed to support important biodiversity values		Yes	Yes	Yes
Criterion 3.3: Institutions or mechanisms exist to govern and manage the site		Criterion not assessed	Yes	Yes
Criterion 3.4: Governance and management of the site achieve or are expected to achieve the in situ conservation of important biodiversity values		Criterion not assessed	Uncertain or partial	Uncertain or partial
Criterion 3.5: In situ conservation of important biodiversity values is expected to be for the long term		Criterion not assessed	Uncertain or partial	uncertain or partial
Criterion 3.6: Governance and management arrangements address equity considerations		Criterion not assessed	Uncertain or partial	Uncertain or partial

**FIGURE 1:**
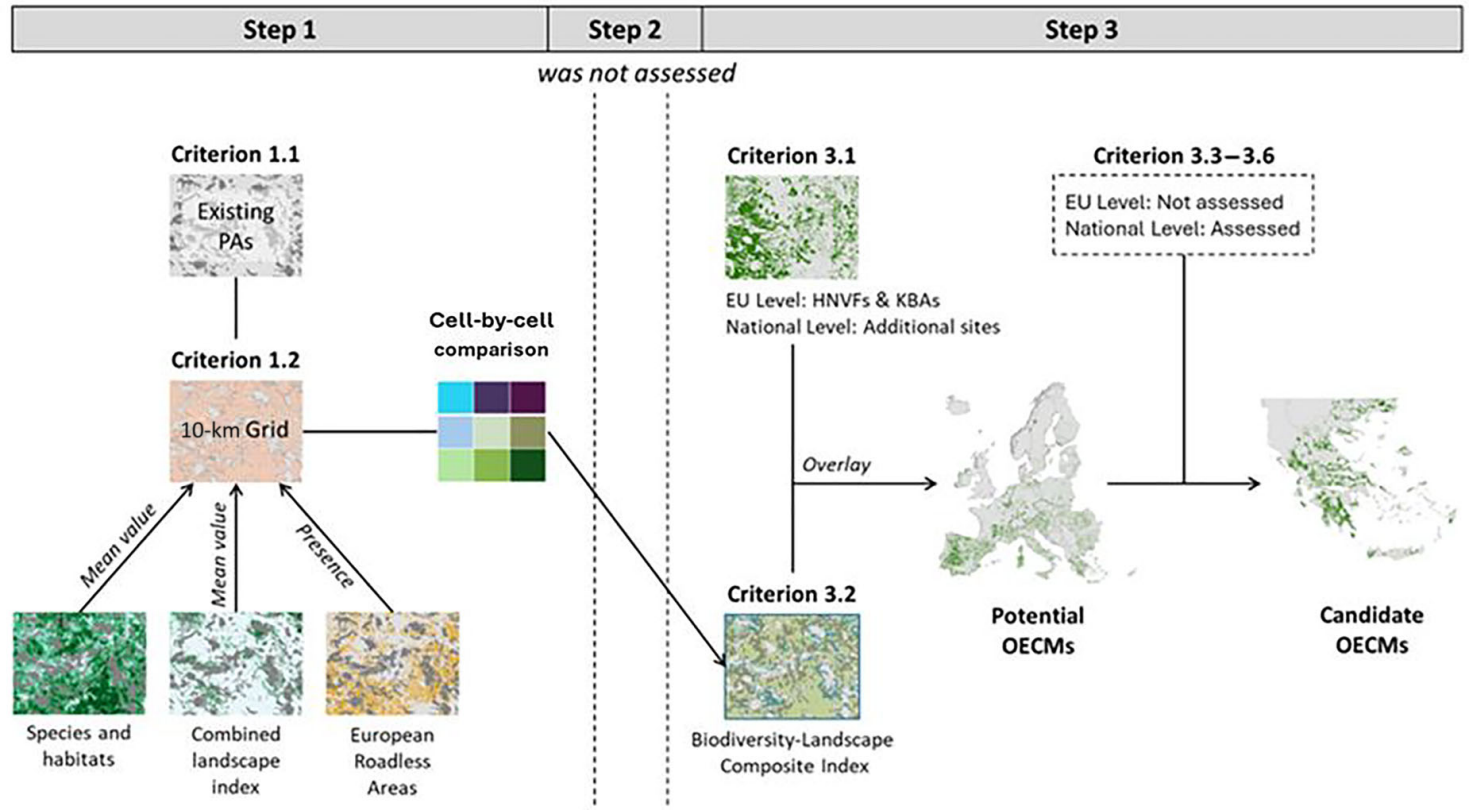
Steps in spatial identification of suitable areas for other effective area‐based conservation measures (OECMs).

In Step 1, two criteria must be met for any area to be considered as an OECM: the site must not already be a designated PA (Criterion 1.1), and there is a reasonable likelihood that the site supports significant biodiversity value (1.2). In Step 2, approval from the governing authority, Indigenous People, local communities, and other right holders is obtained. In Step 3, the site must meet the following six criteria to be confirmed as an OECM: site is a geographically defined area (Criterion 3.1); site supports important biodiversity values (3.2); institutions or mechanisms are in place to govern and manage the site (3.3); governance and management of the site are to achieve or are expected to achieve in situ conservation of important biodiversity values (3.4); important biodiversity values are conserved in situ over the long term (3.5); and equity considerations are addressed in governance and management of the site (3.6). If all criteria are met, the site could be confirmed as an OECM. If the response for some criteria is uncertain or partial, the site is a candidate OECM until further information or characteristics allow for confirmation. If one or more criteria are not met, the site cannot be confirmed as an OECM (Figure 1). Table 1 lists all criteria that were tested at the assessments conducted across spatial scales. Additionally, Appendix S1 includes a step‐by‐step, GIS‐based technical guide detailing the full methodological approach.

### Classification of OECMs

Biodiversity value plays a crucial role in the identification of OECMs, offering a scientific basis for recognizing areas that contribute to long‐term biodiversity conservation. To meet Criterion 1.2, we assessed areas that are likely to support significant biodiversity value with data on species abundance and richness, landscape quality, and degree of human pressures. Species status and trends were estimated based on data at the EU and MS levels. The higher the abundance and richness, the higher the biodiversity value. We included species and habitats designated as priority and/or exhibiting declining trends, as reported in the EU datasets, because our selection focused on areas that meet Criterion 2 of the IUCN's OECM framework, which emphasizes that for OECMs to be recognized, rare, threatened, or declining species should be present (Jonas et al., 2023). The landscape quality was assessed through fragmentation and connectivity indices based on the assumption that less fragmented landscapes support more biodiversity (Valente et al., 2023). Finally, the degree of human pressure was used to indicate that the absence (or low levels) of these pressures indicates a higher (and longer‐term) biodiversity value.

### Capturing biodiversity value

To map areas that can be considered as OECMs in the EU, at the EU27 level, we identified potential OECMs solely based on biodiversity value as determined through initial scoping, whereas at the national level, additional criteria related to governance and management were examined. Three types of OECMs were identified: potential OECMs, areas with potentially high biodiversity value but with undefined boundaries or, in cases where boundaries are defined, lacking governance or management schemes; candidate OECMs, areas with defined boundaries and existing governance or management schemes that could support conservation actions (this differs from the IUCN definition, which requires screening and consent from the relevant governing authority); and confirmed OECMs, areas where biodiversity value has been validated and formally recognized as OECMs. Here, the terms *potential*, *candidate*, and *confirmed OECMs* are based on IUCN's framework, but their meaning has been adapted for the purposes of this article.

The proposed areas were characterized based on biodiversity value and grouped into five classes: very high, high, moderate, low, and very low. In addition, the location of these areas in relation to existing PAs was assessed using a GIS‐based proximity analysis. Each potential OECM was evaluated for its adjacency to PAs. Based on the number of adjacent PAs, we classified spatial configurations as surrounding OECMs (areas that share a boundary with exactly one PA); corridor OECMs (areas adjacent to two or more PAs, potentially serving as ecological linkages); or nonadjacent OECMs (areas with no spatial contact with any existing PA).

### EU‐level assessment

To map areas suitable for OECMs, we used freely available spatial data that cover all EU territory (Table 1). During the screening step, PAs, as reported to the World Database on Protected Areas (WDPA) (UNEP‐WCMC & IUCN, 2025), were identified. This included data on the EU‐wide Natura 2000 PA network and national PA designations as reported through the European inventory of nationally designated areas. The space they occupy was excluded from further processing, as, based on the OECM definition, areas recognized as PAs cannot be OECMs (Criterion 1.1). In the case of Sweden, OECMs reported in the WD‐OECM were not considered because we focused exclusively on identifying suitable areas for OECMs rather than reassessing already reported sites. This may have led to a degree of overlap between the areas we identified and OECMs already recognized in Sweden.

To determine the likelihood that a site supports important biodiversity value (Criterion 1.2), we applied the rationale explained in the section “Capturing biodiversity value.” For the EU‐level assessment, we developed the biodiversity–landscape composite index. The primary data were related to biodiversity, landscape quality, and human threats. Data from the most recent biodiversity reporting by EU Member States of Article 17 habitat types and species (Directive 92/43/EEC) and bird population status and trends from Article 12 (Directive 2009/147/EC) were used to develop a composite spatial layer (layer a) representing occurrence counts per 10‐km grid cell (EC, 2020a) Despite known limitations (Lorilla et al., 2025), these datasets offer full spatial coverage at the scale required for this assessment and were collected through a standardized procedure every 6 years. From these datasets, we selected only those species and habitats designated as priority, exhibiting declining trends, or both. Specifically, we included species for which the field priority was Y (i.e., yes) and the population trend was D (decreasing), as well as habitat types for which priority was Y or the coverage trend was D (decreasing). This selection, although consistent with IUCN guidance on OECM identification, which emphasizes sites that support rare, threatened, or declining biodiversity (Jonas et al., 2023), still omitted species and habitats in poor or unknown condition that may be ecologically significant. All selected species and habitat occurrences were combined without applying normalization or taxonomic weighting. Species and habitat types with greater reporting density may exert proportionally stronger influence on richness patterns and may introduce biases related to uneven data coverage (Appendix S1).

To assess landscape quality, we developed a composite index (layer b). This index was estimated as the average of the normalized values of the forest landscape integrity index (FLII) (Grantham et al., 2020) and the landscape fragmentation index, known as effective mesh size (MESH) (Appendices S2 & S3). The FLII was used to assess forest condition as determined by the degree of anthropogenic modification. It was estimated by integrating four spatially explicit datasets describing the forest extent, pressures from human activities, pressures associated with edge effects, and changes in forest connectivity due to anthropogenic forest loss. MESH expressed the probability of two randomly chosen points in a landscape being connected. The indicator was selected because it indicated areas with well‐connected landscapes. It was estimated by the European Environment Agency (EEA, 2021) and accounted for built area and transportation routes relevant to fragmentation geometry (OpenStreetMaps, 2024) (Jaeger, 2000). Hence, high index values indicated areas with high potential to maintain biodiversity (i.e., low landscape fragmentation and high integrity).

To assess human threats, data on European Roadless Areas were used (Ibisch et al., 2016) (layer c). This dataset was used to indicate areas with limited or no human pressures, which, along with the other indicators, could serve as refuges for wildlife, maintain ecosystem functions, and enhance landscape connectivity. Data on human threats reported under the Habitats and Birds Directive were not used due to data gaps in the reported data (Lorilla et al., 2025; Tsiafouli et al., 2013).

The integration of the biodiversity, landscape fragmentation, and integrity layers was performed through a spatial overlay and classification. Both layers were normalized to a scale of 0–1. Both biodiversity and landscape layers were classified into three categories (low, moderate, high) using the natural breaks (Jenks) method, which identifies optimal class divisions based on natural groupings in the data (Appendix S4). We selected three classes to maintain clarity in the interpretation of the findings, as these classes were subsequently used to generate combined categories. The natural breaks method was selected due to its ability to minimize within‐class variance and better represent the skewed distribution of the indicators, thereby producing more interpretable groupings.

The classified layers were then combined at a 10‐km resolution using a cell‐by‐cell comparison to create a composite index. For each grid cell, the average values of the biodiversity and landscape indices were used to define the five final categories based on the intersection of the two classified layers: very high, both biodiversity and landscape values are high; high, one data type is in high and the other moderate; moderate, both biodiversity and landscape layer values are moderate; low, one data type is low and the other moderate; and, very low, both data types are low.

As a final refinement, any grid cell overlapping with designated roadless areas was reclassified into a higher class to reflect its increased potential for ecological integrity and landscape connectivity. Hence, the biodiversity–landscape composite index identified regions with high biodiversity value (i.e., areas of potential OECMs) (Appendix S5).

We did not consider the consent step because it was not possible to perform a regional spatial analysis of areas that can serve as OECMs. The consent step can only be carried out at the site level with the governing authorities of a potential OECM.

For the full assessment step and to identify geographically defined areas (Criterion 3.1), we considered the two main data sources for which data were available with a full EU MS coverage. We therefore restricted our analysis to areas in the EU that are not designated as PAs but still have clearly defined spatial boundaries, namely, the key biodiversity areas (KBAs) and high nature value farmlands (HNVFs) (Table 1). The KBAs meet standardized criteria for the presence of threatened or geographically restricted biodiversity, ecological integrity, and important biological processes (IUCN, 2016). The HNVFs refer to agricultural areas that support high biodiversity value due to their traditional, low‐intensity farming practices and the presence of seminatural habitats (EEA, 2012; Paracchini et al. 2008).

These spatial data were integrated with the classified biodiversity–landscape composite index. The generated spatial layer combined areas with increased biodiversity value (Criterion 1.2) with areas with clear spatial boundaries. The generated layer included the areas with clear boundaries and different biodiversity conditions. Based on the analysis of Criterion 1.2, areas with high biodiversity value were prioritized into five priority classes (Criterion 3.2). Regarding the governance or management criterion (Criterion 3.3) necessary to support appropriate managerial actions for HNVFs, no such schemes were in place; thus, these areas were characterized as potential OECMs. In the case of KBAs, as defined under the Global Standard for the Identification of KBAs (IUCN, 2016; IUCN SSC/WCPA, 2022), additional criteria have been established, including conditions related to manageability. This implies that it must be possible to implement actions to ensure the conservation of the biodiversity elements for which a KBA has been identified. Accordingly, the KBAs included in this analysis could be considered as candidate OECMs. However, the specific governance or management schemes assigned to the KBAs could not be identified. As a result, similar to HNVFs, the included KBAs were classified as potential OECMs. Criteria 3.4–3.6 were not assessed at this level of analysis due to a lack of homogeneous and unified information at the EU level.

### National‐level assessment

To showcase the applicability of the proposed methodology, we downscaled it to two EU MS, Greece and France, and identified the areas that could be characterized as candidate OECMs. Both countries are recognized as biodiversity hotspots, hosting an estimated 28,752 and 65,773 species, respectively. Both countries established extensive networks of PAs, with terrestrial sites covering 35.01% of Greece and 28.63% of France, including Natura 2000 sites, which cover 27.30 % and 12.98% of each country, respectively (EEA, 2024b). In Greece, the governance and management of PAs are centralized because they are coordinated mainly by the Natural Environment and Climate Change Agency, whereas local management units conduct conservation and monitoring activities within each PA. In France, the governance and management of PAs are pluricentric. The French Office for Biodiversity, a state‐owned public body, coordinates public entities managing PAs. In addition, regional natural parks created by regional authorities develop key actions to manage and preserve landscapes and natural and cultural heritage. The nature reserves in France are federated through an association, registered under the Environment Code since 2016, and work to protect natural heritage.

In the national‐scale adaptation of the proposed approach, a screening step was also conducted following the rationale described above and using the developed biodiversity–landscape composite index. At this level of analysis, we assessed the criteria outlined in Step 3 of the IUCN approach by incorporating additional geographically defined sites beyond KBAs and HNVFs (Criterion 3.1) (Table 1). Portions of these sites that overlapped with existing PAs were excluded from further analyses. All sites were prioritized based on their biodiversity value, estimated using the biodiversity–landscape composite index (Criterion 3.2). However, only those with an existing governance or management mechanism for proposed OECMs were included (Criterion 3.3).

In Greece, additional datasets included archaeological sites located outside PAs, as well as spatially defined forest areas. Archaeological sites are protected by legislation and managed by the local Ephorate of Antiquities. Although their primary aims involve the protection, promotion, and restoration of cultural heritage, biodiversity conservation can occur as a byproduct of these efforts (Kanellou et al., 2024; Panitsa et al., 2022). Forest areas were designated by the Ministry of Environment and Energy and the National Cadastre, which defines forested areas managed by local Forest Service Divisions (FSDs). These divisions are responsible for implementing management plans, determining permitted land uses, and overseeing activities related to biodiversity and forest restoration.

In France, four additional site types were included in the analysis: UNESCO global geoparks, national geological heritage sites, natural spaces, and villages with the *station verte* label. Characterization of these areas was based on national and/or international criteria assessing their natural and cultural value. Governance structures vary for each type—for example, the Ministry of Culture's Directorate‐General for Heritage and Architecture implements the UNESCO Convention for cultural heritage, and the Ministry for Ecological Transition oversees natural heritage. Natural spaces are managed by the Conservatoire d'Espaces Naturels within various frameworks—international, European, national, or local—to meet specific conservation goals for natural heritage.

Despite the existence of institutional mechanisms, full compliance with the criteria outlined in Step 3, particularly those related to governance and management, was not achieved. Specifically, the criteria stating that governance and management of the site achieve or are expected to achieve the in situ conservation of important biodiversity values (Criterion 3.4); that in situ conservation of these values is expected to be long term (Criterion 3.5); and that governance and management arrangements address equity considerations (Criterion 3.6) received responses of uncertain or partial. This was due to uncertainties stemming from political decisions and economic factors, which posed challenges to consistent adherence to the criteria. As a result, the proposed areas were classified as candidate OECMs in the national‐level assessment.

## RESULTS

### Potential OECMs in Europe

**FIGURE 2:**
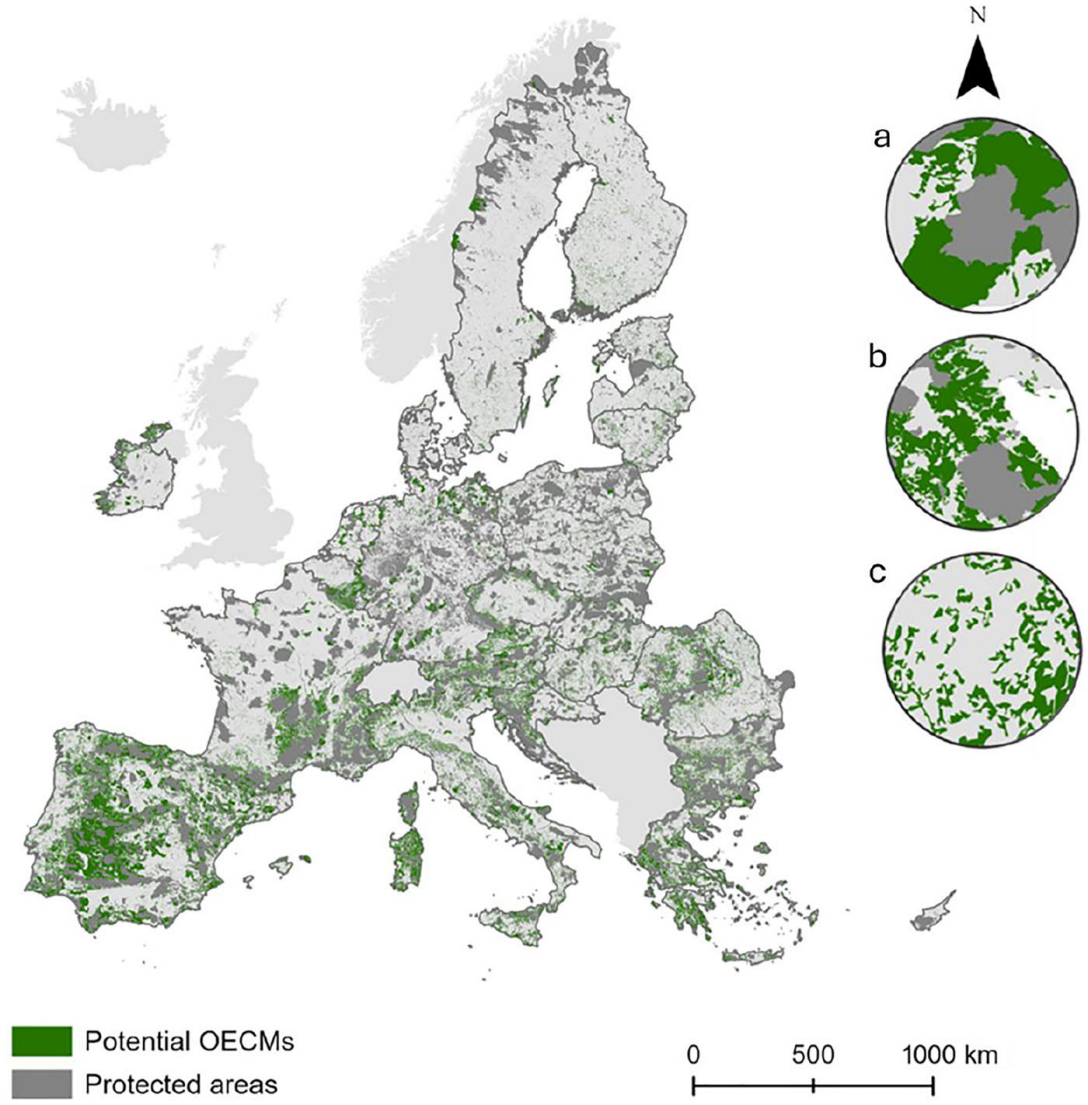
Distribution of potential other effective area‐based conservation measures (OECMs) in Europe and potential OECMs (a) surrounding PAs, (b) as corridors among PAs, and (c) not adjacent to PAs.

Areas suitable for OECMs at the EU level were classified as potential OECMs, given that the full assessment step was not conducted at this level. These areas correspond to 9.68% of the EU's land area (408,849 km^2^) (Appendix S6) with concentrations mostly in central Europe, near the Alps, and in Spain, Italy, and southern France (Figure 2). Specifically, in Spain, Portugal, and Austria, coverage of potential OECMs reaches nearly 20% per country (25% [125,533 km^2^] for Spain, 20% [17,975 km^2^] for Portugal, and 19% [15,732 km^2^] for Austria). At the other extreme, for Denmark and Cyprus, potential OECMs cover only 0.9% (397 km^2^) and 2.3% (216 km^2^), respectively (Figure 2). Regarding the spatial configuration of potential OECMs, the vast majority (approximately 67%) were not adjacent to any PA, whereas 29% were classified as surrounding and 4% as corridors (Appendix S6).

**FIGURE 3:**
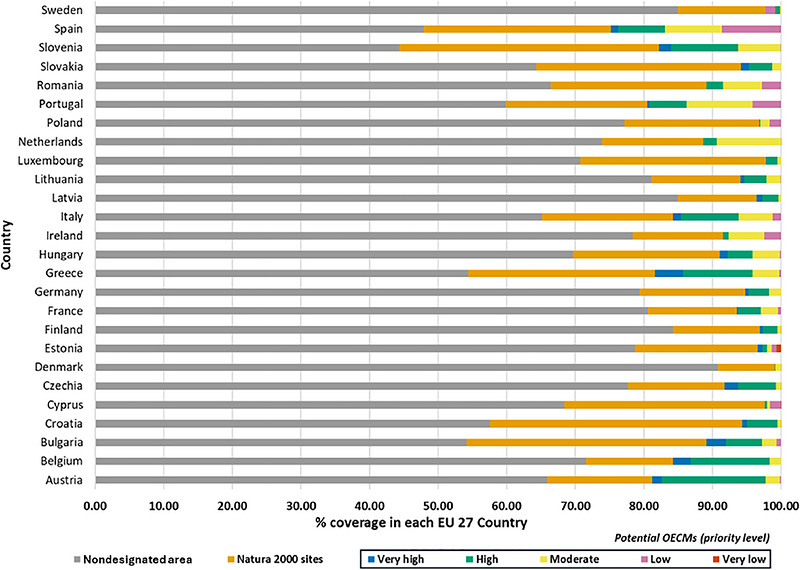
Relative percentage of potential other effective area‐based conservation measures (OECMs) by level of priority based on their biodiversity value and Natura 2000 designation.

These areas, when added to the already established Natura 2000 sites, suggested that the combined total of PAs and OECMs in most countries could potentially cover or even exceed 30% of their total land area. In some countries, for example, Spain, Slovenia, Greece, and Bulgaria, the overall conservation areas could then surpass 45%. In a few countries, primarily in northern Europe, the total coverage of both PAs and OECMs could reach up to 15% of the national territory (Figure 3). Over 50% (204,862 km^2^) of the identified potential OECMs were classified as very high or high priority based on their biodiversity value, 33% (134,393 km^2^) as moderate priority, and 17% (34,796 km^2^) as lower priority (Figure 3; Appendix S6). In Belgium, Croatia, Czechia, Latvia, and Sweden, high‐priority areas for OECMs reached 90% of the total potential OECMs at the national level. In contrast, in Denmark, Hungary, Ireland, Portugal, Romania, and the Netherlands, the majority of potential OECMs were classified as moderate priority (Figure 3; Appendices S6 & S7).

In terms of biodiversity value, the high‐priority potential OECMs identified can potentially conserve an additional 1553 species and 222 habitat types based on the data reported under the Habitats and Birds Directives (Appendix S8). Potential OECMs located in novel areas (i.e., regions where PAs have not been established) covered approximately 3% (436,000 km^2^), 2% (148,000 km^2^), and 5% (920,500 km^2^) of the cumulative monitored areas for species, habitats, and bird species across Europe, respectively (Appendix S8). In terms of species, 379 new bird species could be conserved through the OECM establishment, followed by vascular plants (116 species) and arthropods (82 species). Regarding habitats, forests (51 habitat types) and grasslands (23 habitat types) would predominantly benefit from further protection (Table 2). OECMs have only been reported to the WD‐OECM for Sweden. No other OECMs have been confirmed or included for EU MS, and thus, for most countries, the aforementioned species and habitats were not managed in areas where PAs do not exist.

**TABLE 2:** Number of species and habitats that could be further protected by other effective area‐based conservation measures.

Species group	No. of species	Habitat group	No. of habitat types
Amphibians	40	Bogs, mires, and fens	10
Arthropods	82	Coastal habitat	18
Fish	70	Dune habitat	14
Mammals	70	Forests	51
Mollusks	14	Freshwater habitats	18
Nonvascular plants	16	Grasslands	23
Other invertebrates	4	Heath and scrub	9
Reptiles	49	Rocky habitats	11
Vascular plants	116	Sclerophyllous scrub	8
Birds	412		
Sum of species	461 (36% of species^a^)	Sum of habitat types	162 (70% of habitat types^a^)
Sum of bird species	379 (74% of bird species^b^)		

^a^ Habitats Directive (Article 17, 92/43/EEC): 233 habitat types and 1276 species.

^b^ Birds Directive (Article 12, 2009/147/EC): 508 bird species.

### Candidate OECMs at the national level

Given that the national‐level analysis was further developed to fulfil more criteria within the OECM mapping process, the identified areas met the criteria for being considered candidate OECMs, as they partially fulfilled the requirements of the full assessment step. In Greece, candidate OECMs covered 13.25% (17,503 km^2^) of the total land area, whereas in the EU‐wide assessment, they appeared to cover 18.38% (24,283 km^2^). In France, candidate OECMs covered 2.61% (16,651 km^2^), compared to the EU‐wide assessment where these areas appeared to cover 8.95% (57,721 324 km^2^) (Appendix S9; Figure 3). The candidate OECMs were characterized by very high and high priority for 3.58% (4734 km^2^) of Greece and 1.41% (9.001 km^2^) of France (Appendices S9 & S10).

**FIGURE 4:**
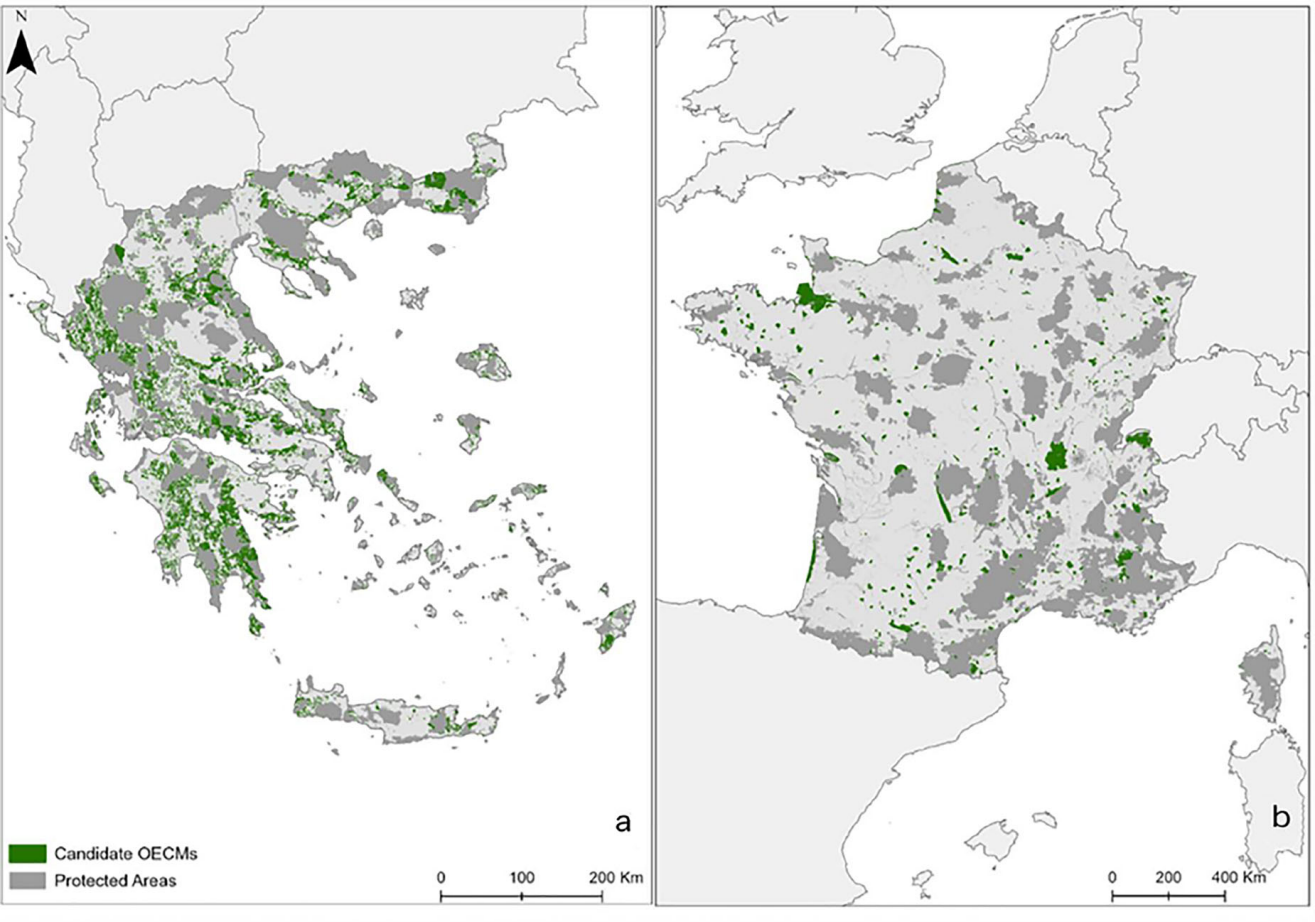
Distribution of candidate other effective area‐based conservation measures (OECMs) and protected areas in (a) Greece and (b) France.

Regarding the spatial distribution of the candidate OECMs, in Greece, more than 80% were classified as nonadjacent to any PA. Similarly, in France, the majority of candidate OECMs were also categorized as nonadjacent (51%), whereas 40% were classified as surrounding PAs (Appendix S9). In Greece, most of the candidate OECMs were located within the Pindus Mountain Range (central and western Greece), a region with a high concentration of PAs (Figure 4). Other clusters of candidate OECMs were found in the mountains of central Peloponnese and the Rhodope Mountain Range in northern Greece. In France, candidate OECMs were primarily located in the southern and eastern parts of the country, in regions with an existing high density of PAs (Figure 4). However, unlike Greece, many areas in France were also distributed in regions with few or no protected sites, particularly in the central and northern parts of the country (Figure 4).

With a focus on high‐value candidate OECMs for biodiversity conservation in Greece, the majority of the nationally monitored species and habitats could be included: 236 species (84%), 95 habitats (93%), and 204 bird species (96%). In France, these numbers were slightly lower, with 237 species (76%), 116 habitats (87%), and 253 bird species (87%) potentially benefiting from complementary protection by OECMs (Appendix S11).

In areas without existing PAs, candidate OECMs in Greece covered approximately 3% (13,600 km^2^) of the cumulative monitored areas for species, 2% (6700 km^2^) for habitats, and 2% (8500 km^2^) for bird species (Appendix S11). These novel areas included 102 species (20% of all species in Greece) and 39 habitat types (38% of all habitat types in Greece) (Table 3). Among individual species groups and habitat types, these OECMs could primarily conserve bird species (36 species), followed by reptiles (30 species) and mammals (11 species). Forest and coastal habitat types were the most represented, with 11 and nine types, respectively, whereas bogs, mires, and fens were not included (Table 3). In France, candidate OECMs in areas without existing PAs covered 1% (29,400 km^2^) of the monitored areas for species, 1% (9200 km^2^) for habitats, and 2% (48,600 km^2^) for bird species across the country (Appendix S11). In these regions, OECMs could additionally conserve 96 species (31% of all species in France) and 39 habitat types (29% of all habitat types in France) (Table 3). Mammals and arthropods, with 29 and 25 species, respectively, would benefit the most from the establishment of OECMs. However, birds and other invertebrates monitored under the Habitat and Bird Directives were not present in identified suitable areas. For habitat types, forest and grassland were the most represented, with 10 and eight types, respectively, whereas no coastal habitat types were included in these suitable areas (Table 3).

**TABLE 3:** Species and habitats that may be further protected by other effective area‐based conservation measures.

Species group	No. of species		Groups of habitat types		No. of habitat types
	Greece	France		Greece	France
Amphibians	8	10	Bogs, mires, and fens	0	2
Arthropods	3	25	Coastal habitats	9	0
Fish	3	8	Dune habitats	5	1
Mammals	11	29	Forests	11	11
Mollusks	0	6	Freshwater habitats	3	7
Nonvascular plants	0	5	Grasslands	4	8
Other invertebrates	1	0	Heath and scrub	1	3
Reptiles	30	5	Rocky habitats	4	3
Vascular plants	10	8	Sclerophyllous scrub	2	4
Birds	45	0			
Sum of species	66 (23% of species^a^)	96 (31% of species^a^)	Sum of habitat types	39 (38% of habitat types^a^)	39 (29% of habitat types^a^)
Sum of bird species	36 (17% of bird species^b^)	–			

^a^ Habitats Directive (Article 17, 92/43/EEC): 102 (Greece) and 134 (France) habitat types and 282 (Greece) and 311 (France) species.

^b^ Birds Directive (Article 12, 2009/147/EC): 213 (Greece) and 292 (France) bird species.

The governance and management scheme of the candidate OECMs in Greece would primarily rely on local FSDs, which are responsible for developing and implementing management plans, as most proposed areas (96% of the candidate OECMs) were characterized as forest. Other proposed areas were in areas recognized as archaeological sites (4% of the candidate OECMs), governed by the local Ephorate of Antiquities. In such cases, biodiversity protection and conservation are not the end goals of management, which falls within the protection, maintenance, and promotion of the monuments and antiquities. Still, in such cases, the protection of these sites might unintentionally safeguard biodiversity hosted within the identified space. Similarly, in France, some proposed areas could be governed and managed by bodies where biodiversity conservation is not central to their mission. For instance, areas proposed for OECMs that fell under the UNESCO Conventions for cultural heritage (58% of the candidate OECMs) might be overseen by the Ministry of Culture's Directorate‐General for Heritage and Architecture. Additionally, candidate OECMs within areas designated as station verte (41% of the candidate OECMs) could be managed by local governments that have achieved and maintained this status.

## DISCUSSION

### Spatial identification of OECMs

At the EU level, the identified areas of potential OECMs cover 9.68% of terrestrial ecosystems. These are areas managed for objectives other than biodiversity conservation, which have significant biodiversity value. Although these areas have not yet been formally assessed for their effectiveness in biodiversity conservation, if they are confirmed to support important biodiversity values, pass all steps for OECM characterization, and implement actions to mitigate impacts on biodiversity, they can contribute to enhancing the conservation efforts of PAs and increasing the societal benefits derived from them.

The spatial distribution and extent of the potential and candidate OECMs support the implementation of the EU BDS and Restoration Regulation (NRR), as management actions beyond existing PAs will be essential (Hermoso et al., 2022). Additionally, OECMs could support Target 3 of the KMGBF by enhancing the overall quality of area‐based conservation networks (CBD, 2022). The methodological approach and the data used facilitate the integration of strategies, actions, and policies developed centrally by the EU and other initiatives. For instance, the inclusion of HNVFs, apart from recognizing their high biodiversity value, aligns with the EU BDS, which aims to restore degraded habitats and promote sustainable agricultural practices. This goal is also supported by the current EU Common Agricultural Policy and the Farm to Fork Strategy (Cuadros‐Casanova et al., 2023; Hermoso et al., 2022). However, the conservation effectiveness of OECMs could change, as in situ biodiversity conservation is, by definition, a byproduct of a management objective. Therefore, alterations in the management regime of an area could also change the conservation effectiveness of OECMs.

Over one third of the mapped potential and candidate OECMs are classified as surrounding or connecting PAs, highlighting their complementary role. The OECM framework provides an opportunity to recognize and support conservation efforts outside of PAs (Jonas et al., 2014). The design of PAs can sometimes be inadequate, either by excluding regions rich in biodiversity (Hilty & Laur, 2021; Kati et al., 2015) or by being too small to support large‐scale ecological processes and the needs of all organisms (Cantú‐Salazar & Gaston, 2010; Zeng et al., 2023). Thus, the additional area offered by OECMs could support ecological processes and functions that current PAs are unable to accommodate (Cantú‐Salazar & Gaston, 2010; Hilty & Laur, 2021; Kati et al., 2015; Zeng et al., 2023). In this regard, the proposed OECMs could offer protection to more than 1000 species and habitats not protected by existing PA networks.

To address connectivity issues among existing PAs, the BDS and the NRR have strengthened the concept of a connected network of PAs, creating a Trans‐European Nature Network (Naumann et al., 2021). The location and landscape characteristics of the proposed OECMs indicate that these areas can play the role of ecological corridors. This would help overcome the connectivity issues of the Natura 2000 network, which is recognized as a common design weakness (Mazaris et al., 2018; Opermanis et al., 2012).

The EU's commitment to both halting biodiversity loss and achieving climate neutrality through the EGD presents a complex challenge (Hermoso et al., 2022; Kati et al., 2021). Although the EGD aims to decouple economic growth from resource use and ensure social justice, its various investment areas, particularly “preserving and restoring ecosystems and biodiversity” and “supplying clean, affordable, and secure energy,”; 2019 can sometimes conflict. On the one hand, the European Commission commits to financially supporting the restoration of ecosystems, recognizing the contribution of nature to economic growth and human health. On the other hand, the decarbonization of energy production is promoted through the development of renewable energy resources (RES), which is crucial to tackling the climate crisis. For example, in Greece, the existing capacity of RES reached 11 gigawatts in 2023, marking a 50% increase compared to 2022 (noting that in 2022, 22.7% of the consumed energy was produced by RES). Considering the goals of the EGD and national energy policy, Greece has set a target to increase renewable energy to 60% of its energy mix by 2030, indicating continued growth in this sector.

However, poorly designed RES, especially in rich biodiversity areas, could pose an obstacle to biodiversity conservation (Hermoso et al., 2022; Kati et al., 2021; Kiesecker et al., 2019). Potential conflict between renewable energy development and the establishment of OECMs exists, where there is overlap in northern Europe (Germany and the Netherlands), the western and northern part of the Iberian Peninsula, and the southern part of Italy (Appendix S10). In Greece, nearly all candidate OECMs overlap existing or planned renewable energy infrastructure (Appendix S12), similar to other regions where OECMs have been proposed, such as in Cyprus (Vogiatzakis & Stavrinides, 2024). Thus, the EU's commitment to both halting biodiversity loss and achieving climate neutrality through the EGD presents a complex challenge, highlighting a need for careful planning and strategic decision‐making (Hermoso et al., 2022; Kati et al., 2021).

### Challenges to OECM establishment

This study provides an analytical framework, based on IUCN's approach (Jonas et al., 2023), for identifying areas that could be confirmed as OECMs. The study focuses on assessing areas solely based on biodiversity value, as determined through initial scoping at the EU level. At the national level, an attempt is made to carry out the full assessment steps of the process by examining criteria related to governance and management. At both levels, we omit the consent section, as it involves a process requiring the engagement of stakeholders, decision makers, and managers, which is beyond the scope of this study. This framework utilizes a composite index that integrates biodiversity data with landscape features, pinpointing areas potentially rich in biodiversity and offering insights for conservation efforts. To be effective, such frameworks should be scalable, user‐friendly, based on accessible data, and adaptable to incorporate new information (Hoffmann, 2022; Margules & Pressey, 2000).

The framework is designed to be straightforward for ecologists and geographers with a basic understanding of geographical data, making it accessible to both researchers and practitioners. Key advantages of the framework are its adaptability and scalability; it has the flexibility to incorporate additional data, allowing it to remain up to date without requiring major reconfigurations. Hence, if data with improved thematic and spatial resolution become available, these can be integrated. As such, this framework operates in a linear manner, where the sequence of data and criteria integration does not impact the result. Instead, the addition of more data enhances the precision of OECM mapping, creating a robust tool for conservation planning that meets the attributes for effectiveness. However, certain methodological choices, such as the division of each input layer into three classes to enhance the operational practicality of the framework, may lead to different spatial configurations and prioritization outcomes if alternative classification approaches or numbers of classes were applied. Consequently, some uncertainty is inherent in the workflow, and such considerations are important when assessing the resulting maps for decision‐making.

However, challenges remain in ensuring data consistency and meeting the IUCN‐defined criteria for OECM confirmation and management. Addressing these challenges requires coordinated efforts at both the EU and national levels. Adequate financial and technical support is essential to ensure compliance and long‐term implementation. Additionally, the availability of appropriate data is critical. This includes information on geographical boundaries and the governance or management regimes of potential OECM sites. Such information is necessary to support site‐level assessments and formal recognition.

Regarding biodiversity, data from the Habitats Directive (Article 17, 92/43/EEC) and Birds Directive (Article 12, 2009/147/EC) offer valuable insights. These datasets help identify areas with rich biodiversity and assess their conservation status. However, despite shared EU‐level guidance for systematic monitoring, individual MS have implemented different monitoring programs. In many cases, they have adapted national schemes to fit their specific needs. This variation can limit consistency and comparability across regions. This has led to inconsistencies in the provided datasets (Ellwanger et al., 2018; Lorilla et al., 2025). Therefore, countries where the biodiversity–landscape composite index values were low might maintain biodiversity levels comparable to those of other EU MS. However, such information may not have been adequately monitored in existing national or European mechanisms. These discrepancies can lead to either the underestimation or overestimation of the status and trends of specific species and habitats (Kudrnovsky et al., 2020). To address these inconsistencies, the EU has been revising national methodologies for monitoring habitat conditions and identifying common variables, with the aim of aligning them with ecosystem accounting methods and requirements (EEA, 2022). Despite these challenges, the dataset remains the most comprehensive at the EU level compared to other sources (Kefalas et al., 2025; Stephenson & Stengel, 2020; Stropp et al., 2016). It is continuously improved and, as the only dataset with consistent funding and regular updates, supports the long‐term monitoring of OECMs.

Our screening also introduces another methodological limitation: we included only species and habitats classified as priority or declining. Although consistent with Criterion 2 of the IUCN OECM framework, this choice excludes stable, data‐deficient, or poorly assessed taxa and may overlook ecologically important areas. Despite the fact that this was done to comply with the IUCN criteria, we recognize that because the prioritization was not tested on the full set of reported biodiversity features, the generalizability of our results remains uncertain. Future work should assess the robustness of the screening using broader inclusion criteria. To address these data gaps, we integrated additional data into the analysis, focusing on other aspects such as landscape structure and ecosystem integrity. Additionally, the use of geographically defined areas estimated with locally specific biodiversity data can help fill these data inconsistencies while also serving as boundaries for the proposed OECMs (Vogiatzakis & Stavrinides, 2024). Given that the formal recognition of OECMs requires the confirmation of biodiversity value, incorporating validated, refined, and context‐specific biodiversity information is essential to support robust, evidence‐based designations.

Beyond the data limitations related to biodiversity, the identification and confirmation of OECMs require the fulfillment of criteria concerning the agreement of local stakeholders, governance, and management of proposed areas, as well as assessment at the site level and on a case‐by‐case basis (Jonas et al., 2023). The inclusion of areas where the primary objective may be beyond biodiversity conservation is a key characteristic of OECMs and a defining attribute that differentiates them from traditional PAs. Hence, without the integration of this characteristic, the proposed methodological framework assesses the areas only for their biodiversity value, like other frameworks for the establishment of PAs (Araújo & Alagador, 2024). Meeting these features and criteria, additional data are needed, which are currently unavailable at the European level. However, at the national scale, context‐specific data may exist and can be integrated into the analysis, allowing for a more detailed identification and mapping of potential OECMs when utilized. Additionally, a preliminary classification based on management objectives is also possible. In the cases of Greece and France, the majority of candidate OECMs are classified as areas where biodiversity conservation is either a byproduct of management objectives, such as archaeological or UNESCO sites, or a secondary objective, as seen with HNVFs or station verte villages. Nevertheless, meeting criteria focusing on achieving or expecting to achieve biodiversity conservation outcomes cannot be guaranteed (Jonas et al., 2023; Marnewick et al., 2021). In this regard, governance and management authorities should address key challenges in OECM confirmation, focusing not only on the actual biodiversity value within the proposed site but also on the governance and management practices of the OECMs.

To address these challenges, Gurney et al. (2021) propose a five‐step procedure that focuses on the governance aspects of OECMs and their efficient management. Specifically, the increased role of local governance concerning equity and rights holders is crucial, while ensuring the security of financial support should be considered by donors and other traditional resources (such as national and local governments and the EC). In this context, one key advantage for the effective confirmation and implementation of OECMs is the concept of polycentric governance (Nagendra & Ostrom, 2012) in which responsibilities are distributed among multiple actors, which can include government agencies, NGOs, private landholders, and local communities. The complex nature of OECMs, along with their multiple values beyond biodiversity conservation, requires a multilevel decision‐making system dedicated to a specific sectoral domain. Multisectoral management approaches can benefit from decentralization and participation of relevant stakeholders and rightsholders from local communities and higher levels of governance (Gurney et al., 2021). Integrating local communities into OECM governance, as seen in areas designated as station verte in France, enhances the effectiveness of OECMs (Danielsen et al., 2024). Local expertise and commitment contribute significantly to biodiversity conservation, whereas communities also benefit directly from the management outcomes.

## Supporting information



Supporting Information
