# Supplementary material for: Spatial identification of areas suitable for other effective area‐based conservation measures in the European Union
Source: Conserv Biol. 2026 Mar 21;40(4):e70263. doi: 10.1111/cobi.70263 (PMC13392769; doi:10.1111/cobi.70263)
Supplement: Supplementary file 1 — Supporting Information [file COBI-40-e70263-s001.docx]

**Supporting Information**

**Appendix S1: Technical Guide for the Identification of Areas Suitable for OECM Recognition**

This appendix is organized into three main sections. The first section (Section 1: Input Dat) describes the input datasets used in the analysis. The second (Section 2: Mapping suitable areas for OECMs) section outlines the three methodological steps adapted from the IUCN’s OECM identification process (Screening, Consent, and Full Assessment). The third section (Section 3: Estimating the added value of OECMs) details the procedure for estimating the additional biodiversity value contributed by the identified potential and candidate OECMs.

To identify and map areas suitable for the establishment of Other Effective area-based Conservation Measures (OECMs), we developed a GIS-based methodology aligned with the IUCN's three-step OECM identification process: **(1) Screening**, **(2) Consent**, and **(3) Full Assessment** (Jonas et al., 2023).

**Section 1: Input Data**

Analyses were performed on a standardized **10 × 10 km grid**, which serves as the spatial unit of analysis. The following datasets were used:

- **Protected Areas**: WDPA data (UNEP-WCMC & IUCN, June 2025)
- **Habitats and Species**: Article 17 (Directive 92/43/EEC) and Article 12 (Directive 2009/147/EC) (EU MS, 2018)
- **Forest Landscape Integrity Index (FLII)**: Determining the degree of anthropogenic modification (Grantham et al. 2020)
- **Landscape Fragmentation Index (MESH)**: Indicated landscape fragmentation (EEA, 2011)
- **European Roadless Areas**: Spatial indicator of ecological integrity (Ibisch et al. 2016)
- **Key Biodiversity Areas (KBAs)** (IUCN, 2016)
- **High Nature Value Farmlands (HNVFs)** (EEA, 2012)

**Additional national datasets:**

**Greece:**

- Archaeological Sites
- National Forest Map

**France:**

- UNESCO Geoparks
- National Geological Heritage Sites
- Natural Spaces
- “Station Verte” Villages

**Section 2: Mapping suitable areas for OECMs**

**Step 1: Screening**

**Output**: A composite index called “Biodiversity-Landscape Composite Index” identifying grid cells with high biodiversity and landscape value.

1. **Mask Protected Areas**
   Remove all grid cells that intersect existing Protected Areas from the analysis.
2. **Filter Biodiversity Records**
   From Article 17 and 12 datasets, select species and habitats where:
   - priority = Y (Yes)
   - and/or population_trend or coverage_trend = D (Decreasing)
3. **Estimate Occurrence Density (Layer A)**
   Count overlapping features of selected habitats and species per grid cell using the *Count Overlapping Features* tool.
4. **Create Landscape Integrity Layer (Layer B)**
   Normalize the **Forest Landscape Integrity Index** and **Effective Mesh Size** to a 0–1 scale. Calculate their average per grid cell.
5. **Normalize and Classify**
   Apply min–max normalization to Layer A and Layer B.
   Then classify each into three Natural Breaks (Jenks) categories:
   - **Low**: 0.00–0.33
   - **Medium**: 0.34–0.66
   - **High**: 0.67–1.00
6. **Composite Index Classification**
   Combine the two classified layers by cell-by-cell comparison to generate five composite categories:
   - **Very High**: Both indices are High
   - **High**: One index is High and one is Medium
   - **Moderate**: Both indices are Medium
   - **Low**: One index is Low and one is Medium
   - **Very Low**: Both indices are Low
7. **Roadless Area Adjustment**
   Grid cells overlapping roadless areas are promoted one class higher, reflecting enhanced ecological integrity and connectivity.

**Step 2: Consent**

This step was not implemented in the current study due to its dependence on local-level stakeholder consultation, which is infeasible at regional scale. Consent assessments can only be performed at the site level.

**Step 3: Full Assessment**

**Output**: Prioritized and spatially classified potential and candidate OECMs.

1. **Overlay Geographical Defined Areas**
   Use spatial intersection to integrate KBAs, HNVFs, and other national datasets with the screening output.
   Assign each area a priority class (Very High, High, Moderate, Low, Very Low).
2. **Candidate OECM Flagging**
   Areas with relevant supporting attributes (e.g., legal recognition, land use status, or ecological function) are flagged as candidate OECMs.
3. **Spatial Configuration Classification**
   Using **Spatial Join (Join One-to-Many)** and summary statistics, evaluate each OECM grid cell’s relationship to existing Protected Areas:
   - **Surrounding**: Touches 1 Protected Area
   - **Corridor**: Touches ≥2 Protected Areas
   - **Non-adjacent**: No spatial contact with any Protected Area

**Section 3: Estimating the added value of OECMs**

**Estimating the added value of the areas for potential and candidate OECMs**

Beyond the identification of the areas suitable for potential OECMs in Europe, a key objective of the study is to evaluate the contribution of these proposed areas to effective biodiversity protection. To fulfil these objectives, we answered two questions: a) How many species and habitats would be further protected by the potential OECMs in areas where PAs do not exist? b) What species and habitats are mostly protected by the potential OECMs? To answer these questions, we utilized species and habitat datasets provided under Article 17 of the Habitats Directive (92/43/EEC) and Article 12 of the Birds Directive (2009/147/EC), respectively. These data, compiled in the most recent report published in 2020, present the results of the third reporting cycle, covering the period 2013–2018.

Species and habitats listed in these directives are monitored and reported systematically every six years by EU Member States, with the European Commission compiling and assessing the data. These datasets extend beyond existing PAs, covering the full natural ranges of species and habitats, and are provided as spatial datasets based on 10 km grid cells. For each species and habitat, a spatial layer indicates its distribution across Europe. As a result, protected species, habitats, and bird species are monitored across cumulative areas of 13,355,100 km², 6,492,500 km², and 19,963,500 km², respectively.

Using a 10 km resolution grid, we identified the cells where PAs and potential OECMs exist, as well as the presence of species and habitats listed in the Habitats and Birds Directives. This allowed us to estimate the number of species and habitats that could be further conserved through the proposed OECMs, both in areas where PAs already exist and where they do not. The R code used to estimate the number of species and habitats that could potentially be further protected by the proposed sites is provided below:

# Purpose: Reproducible statistics for species, habitats, and birds in EU, PAs, and OECMs

# ----------------------------

| ## File Information ----  # Authors: xx  # Date: 08-2025  #  ### Purpose ----  # Estimate OECMs’ biodiversity value  # Global Setting, Libraries ----  # BEFORE:  current_path = rstudioapi::getActiveDocumentContext()$path  setwd(dirname(current_path ))  getwd()  ### Install Packages & Get Libraries ----  # install.packages(c("RODBC","rgdal","sf","dplyr","tidyr","ggpubr","ggsci"))  # library(RODBC)  library(rgdal)  library(sf)  library(dplyr)  #Input data (Birds.EU, Species.EU, and Habitats.EU are the raw data obtained from the European Environment Agency. Birds.OECMs, Species.OECMs, Habitats.OECMs, Birds.PAs, Species.PAs, and Habitats.PAs are the outputs of the GIS intersection process, where the raw biodiversity data were combined with OECMs and PAs.)  Birds.EU <- read.csv("D:/xxx/birds_EU.csv")  Species.EU <- read.csv("D:/xxx/species_EU.csv")  Habitats.EU <- read.csv("D:/xxx/habitats_EU.csv")  Birds.OECMs <- read.csv("D:/xxx/EU_OECMs_Birds.csv")  Species.OECMs <- read.csv("D:/xxx/EU_OECMs_Species.csv")  Habitats.OECMs <- read.csv("("D:/xxx/EU_OECMs_Habitats.csv")  Birds.PAs <- read.csv("D:/xxx/birds_pas.csv")  Species.PAs <- read.csv("D:/xxx/species_pas.csv")  Habitats.PAs <- read.csv("D:/xxx/habitats_pas.csv")  natura.species <- read.csv("D:/xxx/Natura2000_end2021_rev1_SPECIES.csv")  natura.habitats <- read.csv("D:/xxx/Natura2000_end2021_rev1_HABITATS.csv")  other.species <- read.csv("D:/xxx/Natura2000_end2021_rev1_OTHERSPECIES.csv")  ## Calculate statistics ----  uniqCSV <- function(x) { paste(unique(x), sep = ',') }  ### SPECIES - EUROPEAN ----  species.EU <- Species.EU[c(85,15,8,9,11,40)]  #### unique site observations  species.unique <- aggregate(ID_KEY ~ group_, data = as.data.frame(species.EU), FUN=uniqCSV) # aggregate data  for (i in 1:nrow(species.unique)) {  species.unique$count[[i]] <- n_distinct(species.unique$ID_KEY[[i]]) # add count information  }  species.unique$count = as.numeric(species.unique$count) # transform count to numeric type  species.unique.group <- aggregate(count ~ group_, data = species.unique, FUN=sum) # aggregate unique count of species per species group  sum(species.unique.group$count)  ### SPECIES - PAs ====  speciesPAs.df <- Species.PAs[c(109,3,39,32,33,35,64)]  #### unique site observations  speciesPAs.unique <- aggregate(ID_KEY ~ speciescod + group_, data = as.data.frame(speciesPAs.df), FUN=uniqCSV) # aggregate data  for (i in 1:nrow(speciesPAs.unique)) {  speciesPAs.unique$count[[i]] <- n_distinct(speciesPAs.unique$ID_KEY[[i]]) # add count information  }  speciesPAs.unique$count = as.numeric(speciesPAs.unique$count) # transform count to numeric type  speciesPAs.unique.group <- aggregate(count ~ group_, data = speciesPAs.unique, FUN=sum) # aggregate unique count of species per species group  sum(speciesPAs.unique.group$count)  ### SPECIES - OECMs ====  speciesOECMs.df <- Species.OECMs[c(1,2,3,4,5,6,7)]  speciesOECMs.df <- subset(speciesOECMs.df, OECM_Class=="Very high" \| OECM_Class=="High")  #### unique site observations  speciesOECMs.unique <- aggregate(ID_KEY ~ speciescod + group_, data = as.data.frame(speciesOECMs.df), FUN=uniqCSV) # aggregate data  for (i in 1:nrow(speciesOECMs.unique)) {  speciesOECMs.unique$count[[i]] <- n_distinct(speciesOECMs.unique$ID_KEY[[i]]) # add count information  }  speciesOECMs.unique$count = as.numeric(speciesOECMs.unique$count) # transform count to numeric type  speciesOECMs.unique.group <- aggregate(count ~ group_, data = speciesOECMs.unique, FUN=sum) # aggregate unique count of species per species group  sum(speciesOECMs.unique.group$count)  #### SPECIES Unique stats ----  paste0("total=", n_distinct(species.EU$speciescod))  paste0("PAs=", n_distinct(speciesPAs.df$speciescod))  paste0("OECMs=", n_distinct(speciesOECMs.df$speciescod))  #### SPECIES Overlay stats ----  sum(as.numeric(speciesPAs.unique.group$count))  sum(as.numeric(speciesOECMs.unique.group$count))  only.oecms <- anti_join(speciesOECMs.df, speciesPAs.df, by = "ID_KEY")  n_distinct(only.oecms$ID_KEY)  n_distinct(only.oecms$speciescod)  common <- semi_join(speciesPAs.df, speciesOECMs.df, by = "ID_KEY")  n_distinct(common$ID_KEY)  n_distinct(common$speciescod)  oecms.notpas <- common[!(common$speciesnam %in% natura.species$SPECIESNAME) & !(common$SITECODE %in% natura.species$SITECODE),]  n_distinct(oecms.notpas$ID_KEY)  n_distinct(oecms.notpas$speciescod)  oecms.notother <- oecms.notpas[!(oecms.notpas$speciesnam %in% other.species$SPECIESNAME) & !(oecms.notpas$SITECODE %in% other.species$SITECODE),]  n_distinct(oecms.notother$ID_KEY)  n_distinct(oecms.notother$speciescod)  # Get unique values from table1$id and table2$id  unique_values_table1 <- unique(only.oecms$speciesnam)  unique_values_table2 <- unique(oecms.notother$speciesnam)  # Combine unique values from both tables  overall_unique_values <- c(unique_values_table1, unique_values_table2)  # Remove duplicates to get overall unique values  OECMs.value <- n_distinct(overall_unique_values)  #### SPECIES stats per group ----  OECMs.all <- rbind(only.oecms[-1], oecms.notother[-2])  OECMs.unique <- aggregate(speciescod ~ speciesnam + group_, data = as.data.frame(OECMs.all), FUN=uniqCSV) # aggregate data  for (i in 1:nrow(OECMs.unique)) {  OECMs.unique$count[[i]] <- n_distinct(OECMs.unique$speciescod[[i]]) # add count information  }  OECMs.unique$count = as.numeric(OECMs.unique$count) # transform count to numeric type  OECMs.unique.group <- aggregate(count ~ group_, data = OECMs.unique, FUN=sum) # aggregate unique count of species per species group  OECMs.unique.group  sum(OECMs.unique.group$count)  ### HABITATS - EUROPEAN ----  habitats.EU <- Habitats.EU[c(83,11,7,8,9,34)]  #### unique site observations  habitats.unique <- aggregate(ID_KEY ~ group_, data = as.data.frame(habitats.EU), FUN=uniqCSV) # aggregate data  for (i in 1:nrow(habitats.unique)) {  habitats.unique$count[[i]] <- n_distinct(habitats.unique$ID_KEY[[i]]) # add count information  }  habitats.unique$count = as.numeric(habitats.unique$count) # transform count to numeric type  habitats.unique.group <- aggregate(count ~ group_, data = habitats.unique, FUN=sum) # aggregate unique count of species per species group  sum(habitats.unique.group$count)  ### HABITATS - PAs ====  habitatsPAs.df <- Habitats.PAs[c(84,86,12,8,9,10,35)]  #### unique site observations  habitatsPAs.unique <- aggregate(ID_KEY ~ habitatcod + group_, data = as.data.frame(habitatsPAs.df), FUN=uniqCSV) # aggregate data  for (i in 1:nrow(habitatsPAs.unique)) {  habitatsPAs.unique$count[[i]] <- n_distinct(habitatsPAs.unique$ID_KEY[[i]]) # add count information  }  habitatsPAs.unique$count = as.numeric(habitatsPAs.unique$count) # transform count to numeric type  habitatsPAs.unique.group <- aggregate(count ~ group_, data = habitatsPAs.unique, FUN=sum) # aggregate unique count of species per species group  sum(habitatsPAs.unique.group$count)  ### HABITATS - OECMs ====  habitatsOECMs.df <- Habitats.OECMs[c(1,2,3,4,5,6,7)]  # habitatsOECMs.df <- subset(habitatsOECMs.df, priorty=="Y" & cvrg_tr == "D")  habitatsOECMs.df <- subset(habitatsOECMs.df, OECM_Class=="Very high" \| OECM_Class=="High")  #### unique site observations  habitatsOECMs.unique <- aggregate(ID_KEY ~ habitatcod + group_, data = as.data.frame(habitatsOECMs.df), FUN=uniqCSV) # aggregate data  for (i in 1:nrow(habitatsOECMs.unique)) {  habitatsOECMs.unique$count[[i]] <- n_distinct(habitatsOECMs.unique$ID_KEY[[i]]) # add count information  }  habitatsOECMs.unique$count = as.numeric(habitatsOECMs.unique$count) # transform count to numeric type  habitatsOECMs.unique.group <- aggregate(count ~ group_, data = habitatsOECMs.unique, FUN=sum)  sum(habitatsOECMs.unique.group$count)  #### HABITATS Unique stats ----  paste0("total=", n_distinct(habitats.EU$habitatcod))  paste0("PAs=", n_distinct(habitatsPAs.df$habitatcod))  paste0("OECMs=", n_distinct(habitatsOECMs.df$habitatcod))  #### HABITATS Overlay stats ----  sum(as.numeric(habitatsPAs.unique.group$count))  sum(as.numeric(habitatsOECMs.unique.group$count))  only.oecms <- anti_join(habitatsOECMs.df, habitatsPAs.df, by = "ID_KEY")  n_distinct(only.oecms$ID_KEY)  n_distinct(only.oecms$habitatcod)  common <- semi_join(habitatsPAs.df, habitatsOECMs.df, by = "ID_KEY")  n_distinct(common$ID_KEY)  n_distinct(common$habitatcod)  oecms.notpas <- common[!(common$habitatnam %in% natura.habitats$DESCRIPTION) & !(common$SITECODE %in% natura.habitats$SITECODE),]  n_distinct(oecms.notpas$ID_KEY)  n_distinct(oecms.notpas$habitatcod)  oecms.notother <- oecms.notpas[!(oecms.notpas$habitatnam %in% other.species$SPECIESNAME) & !(oecms.notpas$SITECODE %in% other.species$SITECODE),]  n_distinct(oecms.notother$ID_KEY)  n_distinct(oecms.notother$habitatcod)  # Get unique values from table1$id and table2$id  unique_values_table1 <- unique(only.oecms$habitatnam)  unique_values_table2 <- unique(oecms.notother$habitatnam)  # Combine unique values from both tables  overall_unique_values <- c(unique_values_table1, unique_values_table2)  # Remove duplicates to get overall unique values  OECMs.value <- n_distinct(overall_unique_values)  #### HABITATS stats per group ----  OECMs.all <- rbind(only.oecms[c(2,3,4,5,7)], oecms.notother[c(1,3,4,5,6)])  OECMs.unique <- aggregate(habitatcod ~ habitatnam + group_, data = as.data.frame(OECMs.all), FUN=uniqCSV) # aggregate data  for (i in 1:nrow(OECMs.unique)) {  OECMs.unique$count[[i]] <- n_distinct(OECMs.unique$habitatcod[[i]]) # add count information  }  OECMs.unique$count = as.numeric(OECMs.unique$count) # transform count to numeric type  OECMs.unique.group <- aggregate(count ~ group_, data = OECMs.unique, FUN=sum) # aggregate unique count of species per species group  OECMs.unique.group  sum(OECMs.unique.group$count)  n_distinct(common$habitatcod)  oecms.notpas <- common[!(common$habitatnam %in% natura.habitats$DESCRIPTION) & !(common$SITECODE %in% natura.habitats$SITECODE),]  n_distinct(oecms.notpas$ID_KEY)  n_distinct(oecms.notpas$habitatcod)  oecms.notother <- oecms.notpas[!(oecms.notpas$habitatnam %in% other.species$SPECIESNAME) & !(oecms.notpas$SITECODE %in% other.species$SITECODE),]  n_distinct(oecms.notother$ID_KEY)  n_distinct(oecms.notother$habitatcod)  # Get unique values from table1$id and table2$id  unique_values_table1 <- unique(only.oecms$habitatnam)  unique_values_table2 <- unique(oecms.notother$habitatnam)  # Combine unique values from both tables  overall_unique_values <- c(unique_values_table1, unique_values_table2)  # Remove duplicates to get overall unique values  OECMs.value <- n_distinct(overall_unique_values)  #### HABITATS stats per group ----  OECMs.all <- rbind(only.oecms[-4], oecms.notother[-4])  OECMs.unique <- aggregate(habitatcod ~ habitatnam + group_, data = as.data.frame(OECMs.all), FUN=uniqCSV) # aggregate data  for (i in 1:nrow(OECMs.unique)) {  OECMs.unique$count[[i]] <- n_distinct(OECMs.unique$habitatcod[[i]]) # add count information  }  OECMs.unique$count = as.numeric(OECMs.unique$count) # transform count to numeric type  OECMs.unique.group <- aggregate(count ~ group_, data = OECMs.unique, FUN=sum) # aggregate unique count of species per species group  OECMs.unique.group  sum(OECMs.unique.group$count)  ### BIRDS - EUROPEAN ----  birds.EU <- Birds.EU  paste0("total SO=", n_distinct(birds.EU$ID_KEY))  paste0("total US=", n_distinct(birds.EU$speciescod))  ### BIRDS - PAs ====  birdsPAs.df <- Birds.PAs[c(43,3,34,37)]  paste0("PAs SO=", n_distinct(birdsPAs.df$ID_KEY))  paste0("PAs US=", n_distinct(birdsPAs.df$speciescod))  ### BIRDS - OECMs ====  birdsOECMs.df <- Birds.OECMs[c(1,2,3,4,5)]  birdsOECMs.df <- subset(birdsOECMs.df, OECM_Class=="Very high" \| OECM_Class=="High")  paste0("OECMs SO=", n_distinct(birdsOECMs.df$ID_KEY))  paste0("OECMs US=", n_distinct(birdsOECMs.df$speciescod))  #### BIRDS Unique stats ----  paste0("total=", n_distinct(birds.EU$speciescod))  paste0("PAs=", n_distinct(birdsPAs.df$speciescod))  paste0("OECMs=", n_distinct(birdsOECMs.df$speciescod))  #### BIRDS Overlay stats ----  only.oecms <- anti_join(birdsOECMs.df, birdsPAs.df, by = "ID_KEY")  n_distinct(only.oecms$ID_KEY)  n_distinct(only.oecms$speciescod)  common <- semi_join(birdsPAs.df, birdsOECMs.df, by = "ID_KEY")  n_distinct(common$ID_KEY)  n_distinct(common$speciescod)  oecms.notpas <- common[!(common$speciesnam %in% natura.species$SPECIESNAME) & !(common$SITECODE %in% natura.species$SITECODE),]  n_distinct(oecms.notpas$ID_KEY)  n_distinct(oecms.notpas$speciescod)  oecms.notother <- oecms.notpas[!(oecms.notpas$speciesnam %in% other.species$SPECIESNAME) & !(oecms.notpas$SITECODE %in% other.species$SITECODE),]  n_distinct(oecms.notother$ID_KEY)  n_distinct(oecms.notother$speciescod)  # Get unique values from table1$id and table2$id  unique_values_table1 <- unique(only.oecms$speciesnam)  unique_values_table2 <- unique(oecms.notother$speciesnam)  # Combine unique values from both tables  overall_unique_values <- c(unique_values_table1, unique_values_table2)  # Remove duplicates to get overall unique values  OECMs.value <- n_distinct(overall_unique_values) |
| --- |

**Appendix S2.** Ranges of the data used to estimate the “Biodiversity-Landscape Composite Index”

|  | Species & Habitats Art. 17* | Bird Species Art. 12** | FLIII*** | MESH**** |
| --- | --- | --- | --- | --- |
| min | 1 | 1 | 0 | 0.13 |
| max | 122 | 171 | 10 | 100 |

**Species and habitat of Habitats Directive (Article 17, 92/43/EEC), **Birds species of Birds Directive (Article 12, 2009/147/EC)), ***Forest Landscape Integrity Index, **** Landscape fragmentation Index – Effective Mesh Size.*

**Appendix S3: Used formulas**

The following formula used to estimate the composite layer of the conservation status of Article 17 habitat types and species (Directive 92/43/EEC) and bird population status and trends from Article 12 (Directive 2009/147/EC).

$$Composite layer of consrvation status= {Species \& Habitats}_{Art.17}+ {Species}_{Art.12}$$

where: ${Species \& Habitats}_{Art.17}$ is a layer representing the number of species and habitats that have been exhibiting declining trends or have been designated as priorities under Article 17 of Directive 92/43/EEC. ${Species}_{Art.12}$ is the layer of bird species that have been exhibiting declining trends under Article 12 of Directive 2009/147/EC.

The combined landscape index was estimated as the average of the normalized values of the Forest Landscape Integrity Index (FLII) and the Effective Mesh Size (MESH), utilizing the following equations:

$${Combined landscape index}_{(x,y)}= \frac{{FLII}_{\left( norm, x,y \right)}+{MESH}_{(norm, x,y)}}{2}$$

$${FLII}_{\left( norm, x,y \right)}= \frac{{FLII}_{\left( x,y \right)}+{FLII}_{(min, x,y)}}{{FLII}_{\left( max, x,y \right)}+{FLII}_{(min, x,y)}}$$

$${MESH}_{\left( norm, x,y \right)}= \frac{{MESH}_{\left( x,y \right)}+{MESH}_{(min, x,y)}}{{MESH}_{\left( max, x,y \right)}+{MESH}_{(min, x,y)}}$$

where: ${FLII}_{\left( x,y \right)}$ and ${MESH}_{\left( x,y \right)}$ are the values of FLII and MESH in the cell x, y, respectively. ${FLII}_{\left( max, x,y \right)}$, ${FLII}_{\left( min, x,y \right)}$ and ${MESH}_{\left( max, x,y \right)}$, ${MESH}_{(min, x,y)}$ are the maximum and minimum values of FLII and MESH in the cell x, y, respectively. ${FLII}_{\left( norm, x,y \right)}$ and ${MESH}_{(norm, x,y)}$ are the normalized value of the FLII and MESH in the cell x, y, respectively.


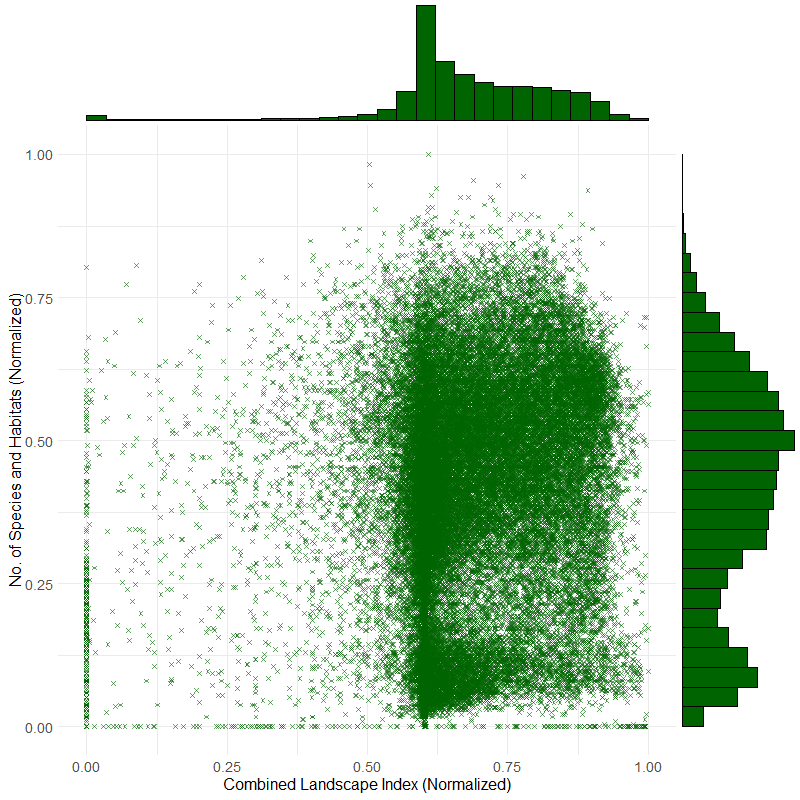


**Appendix S4.** Bivariate plot of the composite layer of the conservation status of Article 17 habitat types and species and the combined landscape index


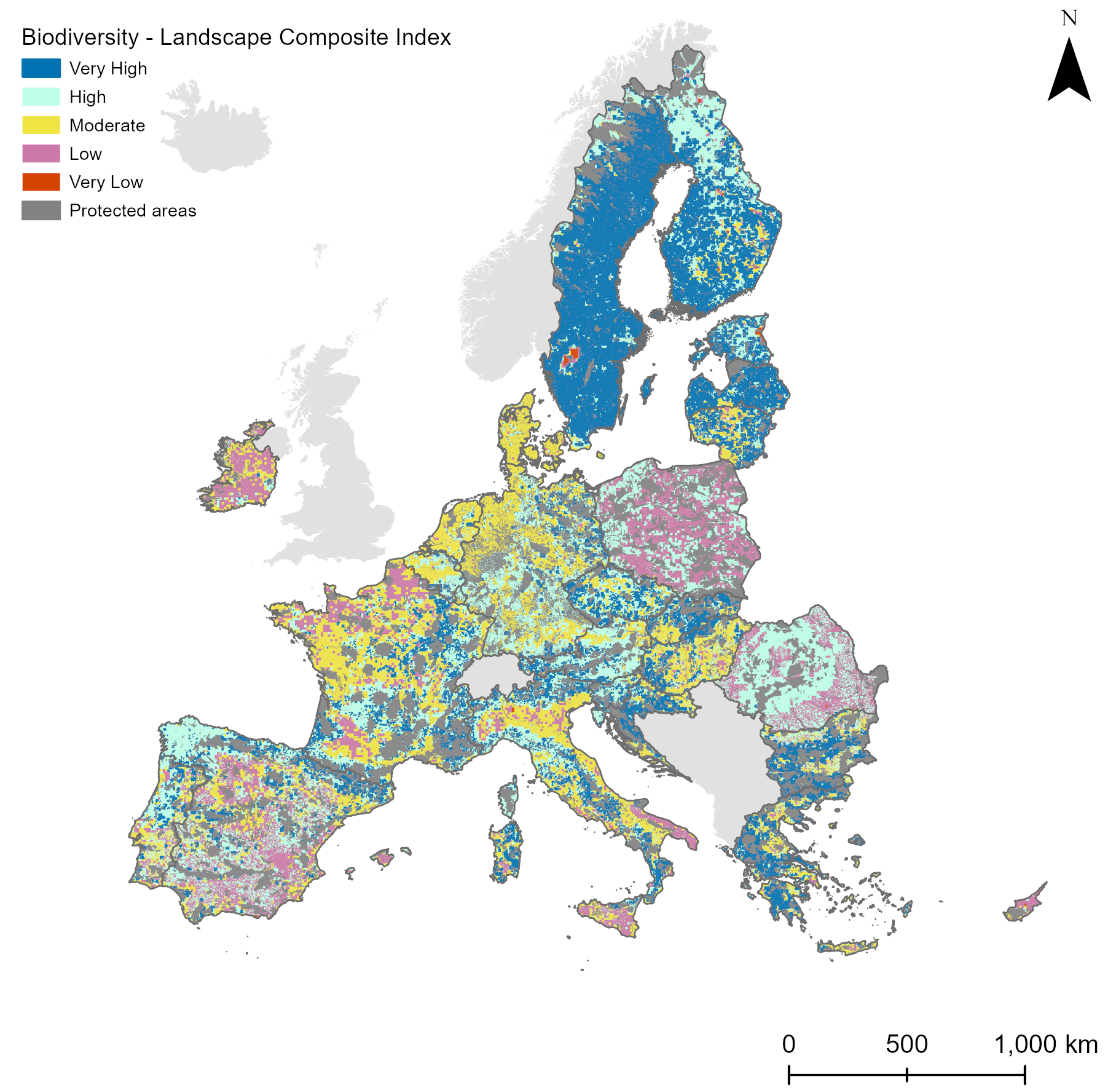


**Appendix S5.** The Biodiversity-Landscape Composite Index. Grid cells are classified into five categories: Very High (both biodiversity and landscape indices ≥ 0.67), High (one ≥ 0.67 and the other between 0.34–0.66), Moderate (both between 0.34–0.66), Low (one ≤ 0.33 and the other between 0.34–0.66), and Very Low (both ≤ 0.33). Grid cells overlapping with roadless areas were promoted one class higher to reflect enhanced ecological integrity and connectivity.

**Appendix S6.** Extent of existing PAs and potential OECMs (per level of priority) with respect to the overall EU territory.

| OECM (level of priority) | Extent (km^2^ & *%*) | Spatial configuration | % of potential OECMs |
| --- | --- | --- | --- |
| Very Low | 50.72 *(0.01%)* | Non-adjacent | 66.96% |
| Low | 69,543 *(17.01%)* | Surrounding | 29.01% |
| Moderate | 134,393 *(32.87%)* | Corridor | 4.03% |
| High | 169,323 *(41.41%)* |  |  |
| Very High | 35,539 *(8.69%)* |  |  |
| Summary | **408,849 *(9.68%)*** |  |  |


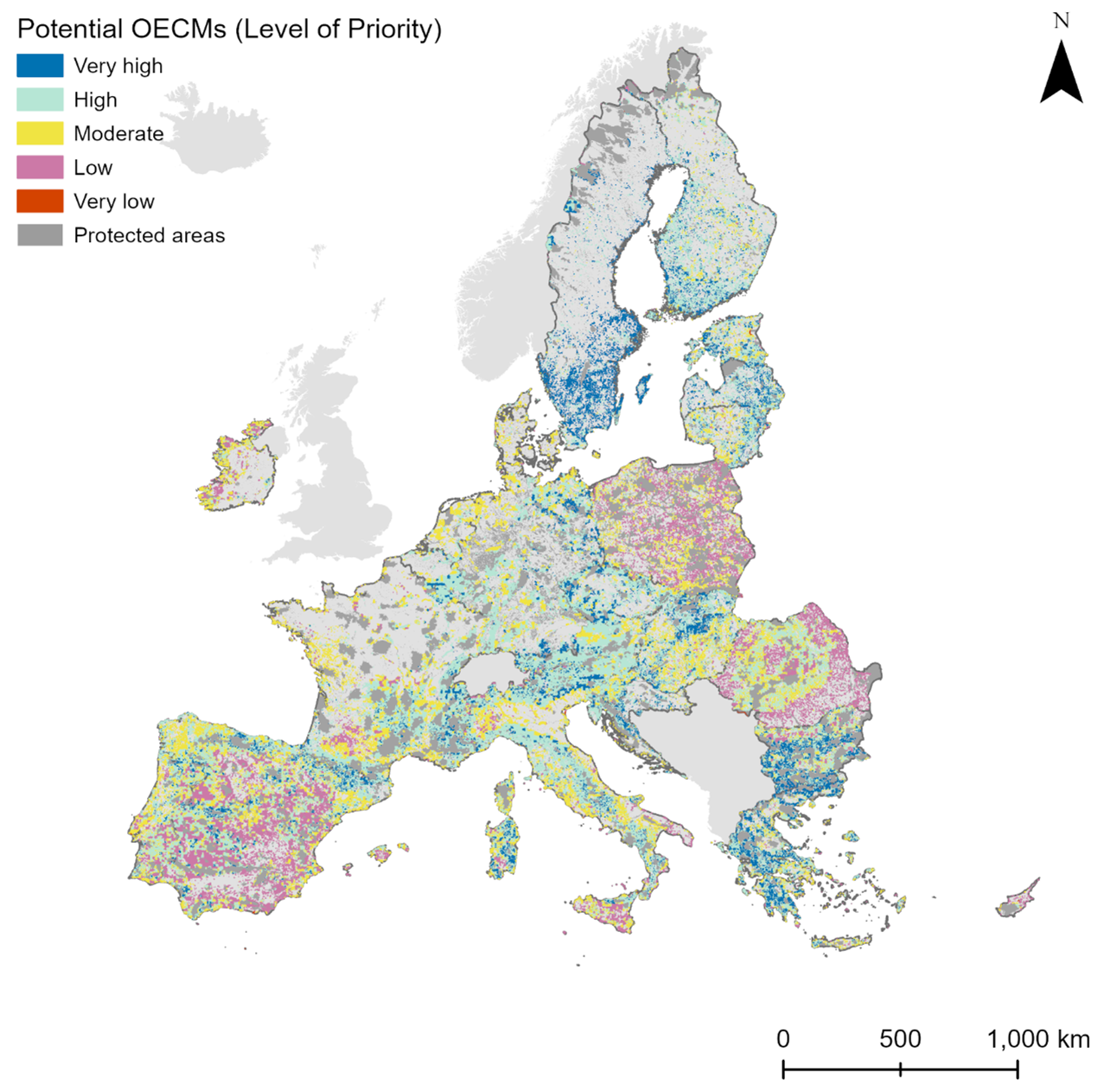


**Appendix S7.** Distribution of the suitable areas for potential OECMs in Europe (Level of Priority). Priority classes follow the Biodiversity–Landscape Composite Index thresholds (Very High, High, Moderate, Low, Very Low) as defined in Figure S5.

**Appendix S8.** The added value of the identified suitable areas for potential OECMs towards biodiversity conservation.

|  |  | Cumulative Monitoring Area  (km2 & % of the total monitoring area) | Species/Habitat |
| --- | --- | --- | --- |
| Species | EU Level | 13,355,100 | 1276 |
|  | PAs | 11,783,800 *(88%)* | 1264 |
|  | OECMs | 8,634,200 *(65%)* | 1045 |
|  | diff OECMs | 436,000 *(3%)* | 418 |
| Habitats | EU Level | 6,492,500 | 233 |
|  | PAs | 5,666,300 *(87%)* | 232 |
|  | OECMs | 3,916,100 *(60%)* | 222 |
|  | diff OECMs | 140,800 *(2%)* | 159 |
| Birds | EU Level | 19,963,200 | 508 |
|  | PAs | 16,788,100 *(84%)* | 486 |
|  | OECMs | 12,626,800*(63%)* | 508 |
|  | diff OECMs | 920,500 *(5%)* | 379 |

**Table S9.** Extent (%coverage of total land area) of candidate OECMs in Greece and France (per biodiversity value).

| OECM (level of priority) | Greece  Extent (km^2^ & %) | France  Extent (km^2^ & %) | Spatial configuration | Greece | France |
| --- | --- | --- | --- | --- | --- |
| Very low | 230.91 *(0.17%)* | 89.79 *(0.01%)* | **Non-adjacent** | 84.75% | 51.46% |
| Low | 15.67 *(0.01%)* | 818.20 *(0.13%)* | **Surrounding** | 14.97% | 40.24% |
| Moderate | 12,522.97 *(9.48%)* | 6,741.29 *(1.06%)* | **Corridor** | 0.28% | 8.30% |
| High | 4,734.30 *(3.58%)* | 7,751.15 *(1.21%)* |  | | |
| Very high | 0 *(0.00%)* | 1,250.70 *(0.20%)* |  |  |  |
| Summary | **17,503.85 *(13.25%)*** | **16,651.21 (2.61*%)*** |  |  |  |


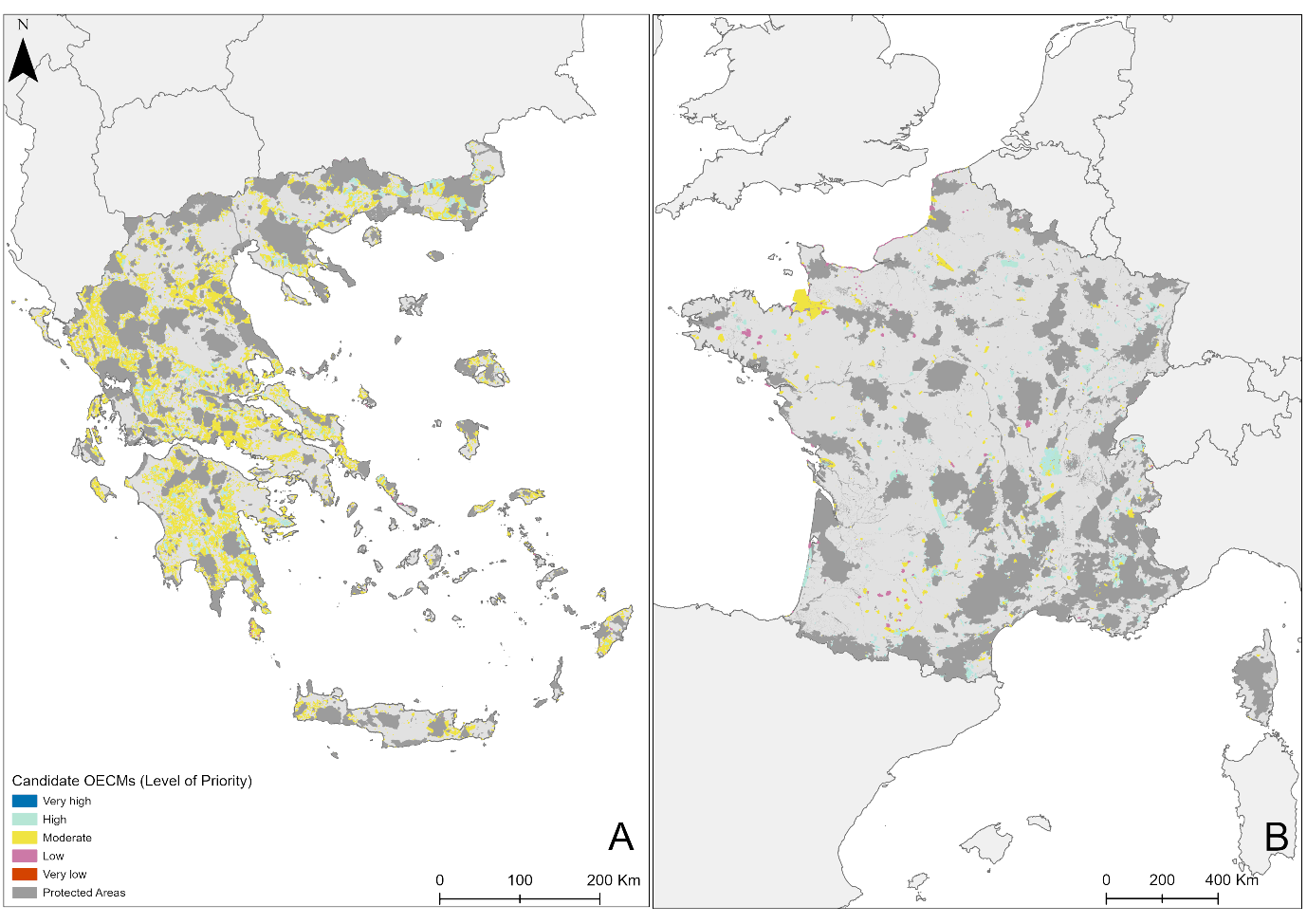


**Appendix S10.** Distribution of the suitable areas for potential OECMs in Greece (A) and France (B) (Level of Priority). Priority classes follow the Biodiversity–Landscape Composite Index thresholds (Very High, High, Moderate, Low, Very Low) as defined in Figure S5.

**Appendix S11.** The added value of the identified suitable areas for potential OECMs towards biodiversity conservation.

|  |  | Cumulative Monitoring Area  (km2 & % of the total monitoring area) | | | Species/Habitat | | |
| --- | --- | --- | --- | --- | --- | --- | --- |
|  |  | **Greece** | **France** | **Greece** | | **France** |  |
| Species | National level | 479,700 | 2,175,100 | 282 | | 311 |  |
|  | PAs | 457,300 *(95%)* | 1,921,100 *(88%)* | 282 | | 311 |  |
|  | OECMs | 297,100 *(62%)* | 523,900 *(24%)* | 236 | | 237 |  |
|  | diff OECMs | 13,600 *(3%)* | 29,400 *(1%)* | 60 | | 69 |  |
| Habitats | National level | 329,200 | 923,000 | 102 | | 134 |  |
|  | PAs | 315,400 *(96%)* | 852,500 *(92%)* | 102 | | 134 |  |
|  | OECMs | 173,600 *(53%)* | 281,600*(31%)* | 95 | | 116 |  |
|  | diff OECMs | 6,700 *(2%)* | 9,200 *(1%)* | 39 | | 39 |  |
| Birds | National level | 570,900 | 2,715,100 | 213 | | 292 |  |
|  | PAs | 545,600 *(96%)* | 2,299,000 *(85%)* | 213 | | 292 |  |
|  | OECMs | 298,000 *(52%)* | 671,000 *(25%)* | 204 | | 253 |  |
|  | diff OECMs | 8,500 *(2%)* | 48,600 *(2%)* | 36 | | 0 |  |


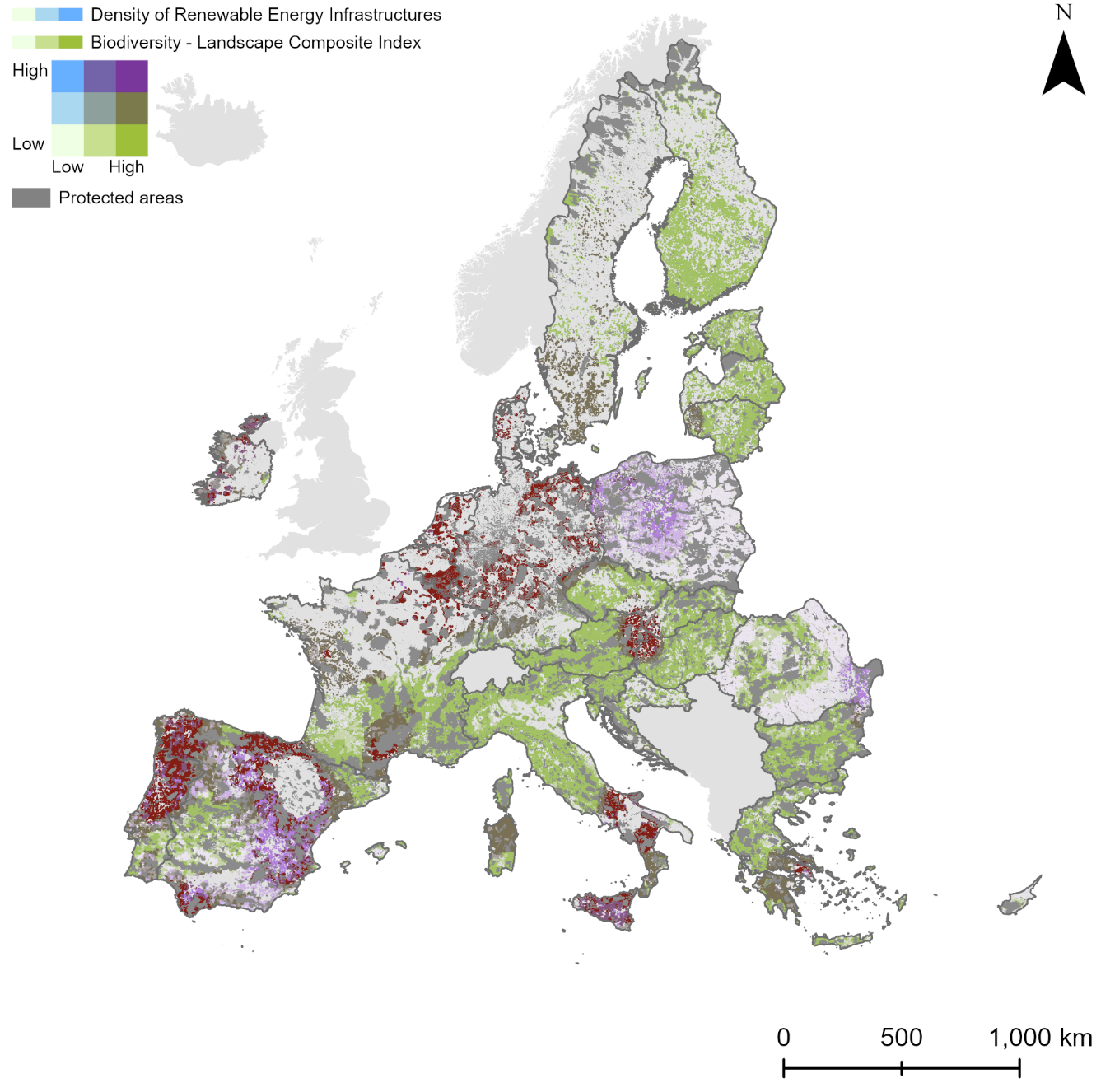


**Appendix S12.** Spatial matches between suitable areas for potential OECMs and Renewable Energy Infrastructures. Renewable energy infrastructure data were sourced from the Open Street Map dataset (OSM, 2025), including wind farms facilities. Density was calculated as the total number of infrastructure features per 10 km × 10 km grid cell and classified into three equal-interval classes: Low, Medium, and High. For visual consistency, the OECM layer was reclassified into three categories by merging “Very High” and “High” into a single High class, and “Low” and “Very Low” into a single Low class. This map is presented to illustrate potential spatial co-existence between potential OECMs and renewable energy infrastructures.
